# Systematic analysis of dark and camouflaged genes reveals disease-relevant genes hiding in plain sight

**DOI:** 10.1186/s13059-019-1707-2

**Published:** 2019-05-20

**Authors:** Mark T. W. Ebbert, Tanner D. Jensen, Karen Jansen-West, Jonathon P. Sens, Joseph S. Reddy, Perry G. Ridge, John S. K. Kauwe, Veronique Belzil, Luc Pregent, Minerva M. Carrasquillo, Dirk Keene, Eric Larson, Paul Crane, Yan W. Asmann, Nilufer Ertekin-Taner, Steven G. Younkin, Owen A. Ross, Rosa Rademakers, Leonard Petrucelli, John D. Fryer

**Affiliations:** 1 Department of Neuroscience, Mayo Clinic, Jacksonville, FL 32224 USA; 2 Mayo Clinic Graduate School of Biomedical Sciences, Jacksonville, FL 32224 USA; 3 Department of Biology, Brigham Young University, Provo, UT 84602 USA; 4 Department of Pathology, University of Washington, Seattle, WA 98195 USA; 5 Department of Medicine, University of Washington, Seattle, WA 98195 USA; 6 Department of Health Sciences Research, Mayo Clinic, Jacksonville, FL 32224 USA; 7 Department of Neurology, Mayo Clinic, Jacksonville, FL 32224 USA

**Keywords:** Camouflaged genes, Dark genes, Long-read sequencing, Pacific Biosciences (PacBio), Oxford Nanopore Technologies (ONT), 10x Genomics, Alzheimer’s Disease Sequencing Project (ADSP), CR1, APOE

## Abstract

**Background:**

The human genome contains “dark” gene regions that cannot be adequately assembled or aligned using standard short-read sequencing technologies, preventing researchers from identifying mutations within these gene regions that may be relevant to human disease. Here, we identify regions with few mappable reads that we call dark by depth, and others that have ambiguous alignment, called camouflaged. We assess how well long-read or linked-read technologies resolve these regions.

**Results:**

Based on standard whole-genome Illumina sequencing data, we identify 36,794 dark regions in 6054 gene bodies from pathways important to human health, development, and reproduction. Of these gene bodies, 8.7% are completely dark and 35.2% are ≥ 5% dark. We identify dark regions that are present in protein-coding exons across 748 genes. Linked-read or long-read sequencing technologies from 10x Genomics, PacBio, and Oxford Nanopore Technologies reduce dark protein-coding regions to approximately 50.5%, 35.6%, and 9.6%, respectively. We present an algorithm to resolve most camouflaged regions and apply it to the Alzheimer’s Disease Sequencing Project. We rescue a rare ten-nucleotide frameshift deletion in CR1, a top Alzheimer’s disease gene, found in disease cases but not in controls.

**Conclusions:**

While we could not formally assess the association of the *CR1* frameshift mutation with Alzheimer’s disease due to insufficient sample-size, we believe it merits investigating in a larger cohort. There remain thousands of potentially important genomic regions overlooked by short-read sequencing that are largely resolved by long-read technologies.

**Electronic supplementary material:**

The online version of this article (10.1186/s13059-019-1707-2) contains supplementary material, which is available to authorized users.

## Background

Researchers have known for years that large, complex genomes, including the human genome, contain “dark” regions—regions where standard high-throughput short-read sequencing technologies cannot be adequately assembled or aligned—thus preventing our ability to identify mutations within these regions that may be relevant to human health and disease. Some dark regions are what we term “dark by depth” (few or no mappable reads), while others are what we term “dark by mapping quality” (reads aligned to the region, but with a low mapping quality). Regions that are dark by depth may arise because the region is inherently difficult to sequence at the chemistry level (e.g., high GC content [1, 2]), essentially eliminating sequencing reads from that region altogether. Other dark regions arise, not because the sequencing is inherently problematic, but because of bioinformatic challenges. Specifically, many dark regions arise from duplicated genomic regions, where confidently aligning short reads to a unique location is not possible; we term these regions as “camouflaged”. These camouflaged regions are generally either large contiguous tandem repeats (e.g., centromeres, telomeres, and other short tandem repeats), or a larger specific DNA region that has been duplicated (e.g., a gene duplication) either in tandem or in a more distal genome region. In fact, many genes in the human genome were duplicated over evolutionary time and are still transcriptionally and translationally active (e.g., heat-shock proteins) [3–9], while others have been duplicated, but are considered inactive (i.e., pseudogenes). Regardless of whether the duplication is active, however, any genomic region that has been nearly identically duplicated and is large enough to prevent sequencing reads from aligning unambiguously will be “dark”, because the aligner cannot determine which genomic region the read originated from.

When confronted with a read that aligns equally well to two or more camouflaged regions (commonly known as multi-mapping reads [2, 10]), standard next-generation sequence aligners, such as the Burrows-Wheeler Aligner (BWA) [11–13], randomly map the read to one of the regions and assign a low mapping quality. For BWA, specifically, reads that cannot be uniquely mapped are generally assigned a mapping quality (MAPQ) of 0; though, in certain paired-end sequencing scenarios, BWA will assign a high mapping quality if the read mate is confidently mapped nearby (i.e., within the estimated insert-size length).

Recent work has characterized camouflaged regions, in part, including a study that demonstrates how this issue affects all standard RNA-Seq analyses [10] and another that quantifies the number of nucleotides in human reference GRCh38 that are dark from mapping quality of 0 (camouflaged regions), based on 1000 Genome Project data [2]. Robert and Watson demonstrated that expression for 958 genes were either over- or under-represented because of multi-mapping reads across 12 different RNA-Seq processing methods, and no method was immune to the problem [10]. They also demonstrated that many of these genes are directly implicated in human disease. Zheng-Bradley et al. recently re-aligned genomes from the 1000 Genomes Project to GRCh38, and, among other findings, generally demonstrated the breadth of multi-mapping reads across the genome [2]. These data characterize the general problem and report specific genes affected by this issue.

Here, we systematically analyze dark and camouflaged genes to more fully characterize the problem, and we highlight many disease-relevant genes that are directly implicated in neurological diseases and conditions such as Alzheimer’s disease, autism spectrum disorder, amyotrophic lateral sclerosis (ALS), spinal muscular atrophy (SMA), and others. We also show that linked-read and long-read sequencing technologies substantially reduce the number of dark and camouflaged regions, and we present a method to address camouflaged regions, even in standard short-read sequencing data. As a proof of concept, we applied our method to the Alzheimer’s Disease Sequencing Project (ADSP) data and identified a rare, ten-nucleotide frameshift deletion in the C3b and C4b binding domain of *CR1*, a top Alzheimer’s disease gene [14–22], that is present in five ADSP cases but zero controls. The ADSP is not large enough to statistically assess association between the *CR1* frameshift mutation and Alzheimer’s disease, but this warrants further investigation.

## Results

To quantify the number of dark and camouflaged regions in standard short-read whole-genome sequencing data, we obtained whole-genome sequencing data for ten unrelated males from the Alzheimer’s Disease Sequencing Project (ADSP) and scanned each sample for dark and camouflaged regions, averaging across all ten samples; we only used data from males in this study so we could also assess dark and camouflaged regions on the Y chromosome because large portions of the Y chromosome are dark. We ignored incomplete genomic regions (e.g., centromeres). For most of our analyses, we then limited the dark and camouflaged regions to known gene bodies, based on annotations from build 93 of the GRCh38 human reference genome, excluding alternate contig assemblies. For comparison, we performed the same analyses on GRCh38 including alternate contig assemblies, and on build 87 of the Ensembl GRCh37 human reference genome [23]. All ten samples were sequenced using standard Illumina whole-genome sequencing with 100-nucleotide read lengths, where median genome-wide read depths ranged from 33.0x to 45.0x coverage, with an overall median of 37.5x. We performed the same analyses on ten unrelated males from the 1000 Genomes Project [24] that were sequenced using Illumina whole-genome sequencing with 250-nucleotide read lengths, where median genome-wide read depths ranged from 30.0x to 61.0x coverage, with an overall median of 58.5x. Similarly, we assessed how well linked-read or long-read sequencing technologies from 10x Genomics (52x median coverage), PacBio (50x median coverage), and ONT (down-sampled to ~ 52x median coverage) resolve dark and camouflaged regions. Although we were only able to obtain a single high-depth male genome for each long-read technology, we believe our results are a reasonable estimate for how well each technology addresses dark and camouflaged regions. Larger sequencing studies will further clarify our results.

**Fig. 1:**
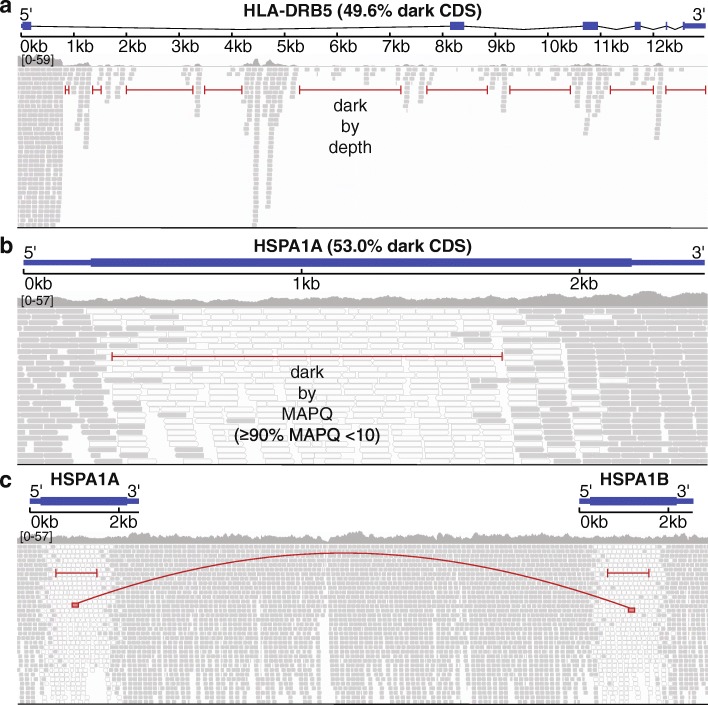
Genomic regions may be “dark” by depth or mapping quality, many of which are “camouflaged”. Large, complex genomes are known to contain “dark” regions where standard high-throughput short-read sequencing technologies cannot be adequately assembled or aligned. We split these dark regions into two types: (1) dark because of low depth and (2) dark because of low mapping quality (MAPQ), which are mostly “camouflaged”. **a** *HLA-DRB5* encodes a Major Histocompatibility Complex protein that plays an important role in immune response and has been associated with several diseases, including Alzheimer’s disease. It is well known to be dark (low depth); specifically, when performing whole-genome sequencing using standard short-read sequencing technologies, an insufficient number of reads align, preventing variant callers from assessing mutations. We calculated sequencing depth across *HLA-DRB5* for ten male samples from the Alzheimer’s Disease Sequencing Project (ADSP) that were sequenced using standard Illumina whole-genome sequencing with 100-nucleotide read lengths. Approximately 63.5% (49.6% of coding sequence) of *HLA-DRB5* is dark by depth (≤ 5 aligned reads; indicated by red lines). **b** *HSPA1A* is a heat-shock protein from the 70-kilodalton (kDa) heat-shock protein family and plays an important role in stabilizing proteins against aggregation. *HSPA1A* is dark because of low mapping quality (MAPQ < 10 for ≥ 90% of reads at a given position). Approximately 41.1% (53.0% coding sequence) of *HSPA1A* is dark by mapping quality (indicated by red line). Dark gray bars indicate sequencing reads with a relatively high mapping quality, whereas white bars indicate reads with a low mapping quality (MAPQ = 0). **c** Many genomic regions that are dark because of mapping quality arise because they have been duplicated in the genome, which we term “camouflaged” (or “camo genes”). When confronted with a read that aligns equally well to more than one location, standard sequence aligners randomly assign the read to one location and give it a low mapping quality. Thus, it is unclear from which gene any of the reads indicated by white bars originated from. *HSPA1A* and *HSPA1B* are clear examples of camouflaged genes arising from a tandem duplication. The two genes are approximately 14 kb apart and approximately 50% of the genes are identical

We consider a region “dark” for one of two reasons: (1) insufficient number of reads aligned to the genomic region (dark by depth) and (2) reads aligned to the region, but with insufficient mapping quality for a variant caller to identify mutations in the region (dark by mapping quality). Specifically, we define regions that are dark by depth as those with ≤ 5 aligned reads (Fig. 1a) and regions that are dark by mapping quality as those where ≥ 90% of aligned reads have a mapping quality (MAPQ) < 10 (Fig. 1b). Defining dark-by-depth regions as those with ≤ 5 reads is a relatively strict cutoff and likely underestimates the number of dark regions because 20 to 30 reads is often considered a reasonable minimum to confidently identify heterozygous mutations; overall median read depth is an important factor, however, and we believe a strict cutoff provides a more conservative estimate. We used a mapping quality threshold < 10 to define regions that are dark by mapping quality because that is the standard cutoff used in the Genome Analysis ToolKit (GATK) [25]. Camouflaged regions are those that are dark by mapping quality because the region has been duplicated in the genome (Fig. 1c). We identified sets of camouflaged regions (regions camouflaged by each other) using BLAT [26], where we required at least 98% sequence identity for two regions to be included in the same set.

### Standard short-read sequencing leaves 36,794 dark regions across 6054 gene bodies, including protein-coding exons from 748 genes

**Fig. 2:**
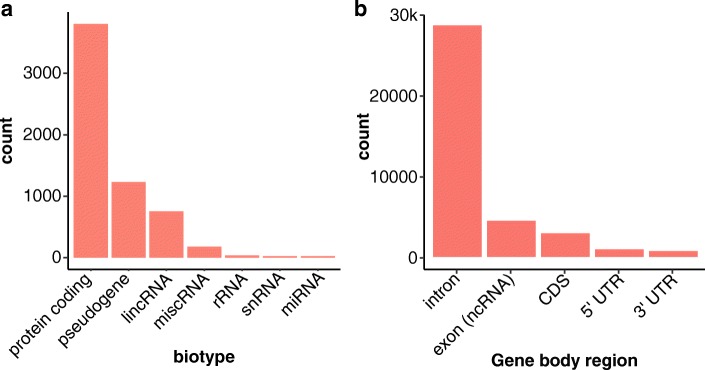
Many dark regions involve protein-coding gene regions. We identified 36,794 dark regions (> 15 million nucleotides) in 6054 gene bodies that were either dark by depth or dark by mapping quality. **a** Stratifying the gene bodies by GENCODE biotype, 3804 gene bodies were protein coding, 1232 were pseudogenes, and 753 were long intergenic non-coding RNAs (lincRNA). **b** Of all 36,794 dark regions, 27,982 were intronic, 4351 were in ncRNA exons, 2855 were in protein-coding exons (CDS), 908 were in 5′UTR regions, and 698 were in 3′UTR regions. Any dark region that spanned a gene element boundary (e.g., intron to exon) was split into separate dark regions

Using whole-genome Illumina sequencing data (100-nucleotide read lengths) from ten unrelated males, we identified 36,794 dark regions (> 15 million nucleotides) in 6054 gene bodies (based on Ensembl GRCh38 build 93 gene annotations) that were either dark by depth or dark by mapping quality (Table 1; Additional file 1: Figure S1a; Additional file 2: Table S1; Additional file 3: Table S2). Stratifying the gene bodies by GENCODE biotype [27], 3804 gene bodies were protein coding, 1232 were pseudogenes, and 753 were long intergenic non-coding RNAs (lincRNA; Fig. 2a). Of all 36,794 dark gene-body regions, 27,982 were intronic, 4351 were in non-coding RNA exons (e.g., lincRNAs and pseudogenes), 2855 were in protein-coding exons (CDS), 908 were in 5′UTR regions, and 698 were in 3′UTR regions (Fig. 2b; Additional file 2: Table S1). Any dark region that spanned a gene element boundary (e.g., intron to exon) was split into separate dark regions. Of the 6054 gene bodies, 527 (8.7%) were 100% dark, 1608 (26.6%) were at least 25% dark, and 2128 (35.2%) were at least 5% dark (Additional file 1: Figure S1b; Additional file 2: Table S1). In intragenic regions, there were a total of 68.7 million nucleotides that were dark in 84,174 regions, totaling 83.8 million dark nucleotides and 90,228 regions, genome wide (Table 1). We also found that aligning GRCh38+alt increased the number of dark nucleotides > 3 times compared to GRCh37.

**Table 1:** Dark and camouflaged regions vary by genome build. We identified dark and camouflaged regions throughout the genome for three different builds, including GRCh37, GRCh38, and GRCh38+alt, across five different sequencing technologies (or read lengths for Illumina). Specifically, we measured dark regions for Illumina based on 100-nucleotide read lengths, Illumina based on 250-nucleotide read lengths, 10x Genomics, PacBio, and Oxford Nanopore Technologies (ONT). Here, the counts for dark and camouflaged regions are combined. We found that the number of dark regions and nucleotides, both within gene bodies (represented as GB in the table) and outside gene bodies, varies dramatically by build and technology. Overall, each technology has its respective strengths. GRCh38 including alternate contigs has > 3x more dark nucleotides than GRCh37, and more than 2x more dark regions. Results presented throughout the manuscript are based on GRCh38 (in gray)

Dark regions	GRCh37					GRCh38					GRCh38+alt		
	il100	il250	10x	PacBio	ONT	il100	il250	10x	PacBio	ONT	il100	PacBio	ONT
Non-GB nucs.	22.4M	15.7M	5.4M	11.1M	6.7M	68.7M	42.5M	57.0M	56.8M	52.1M	88.4M	69.5M	59.1M
Non-GB regs.	38,931	16,247	17,481	10,615	13,441	84,174	54,418	20,650	20,276	23,613	91,263	35,136	25,682
GB nucs.	16.3M	11.4M	4.2M	6.7M	3.7M	15.1M	12.2M	4.3M	6.4M	3.3M	41.6M	26.9M	16.2M
GBs	5857	4424	3828	2095	4454	6054	4227	3993	2170	4465	7396	3332	4465
Protein-coding	3792	2814	2845	1251	3464	3804	2437	2875	1275	3406	4291	1741	3041
Pseudogenes	1134	955	454	483	417	1232	1080	518	474	425	1701	876	668
lincRNAs	732	492	398	254	476	753	513	459	284	546	920	417	529
Others	199	163	131	107	97	265	197	141	137	88	484	298	227
GB regions	37,874	20,030	15,076	9729	9757	36,794	21,052	14,878	8999	8701	59,703	29,302	20,657
Intronic	28,751	13,971	11,700	6632	8000	27,982	14,405	11,322	6126	7371	41,219	18,842	14,029
ncRNA exons	4188	2799	1052	1734	959	4351	3396	1216	1738	878	6589	3573	2117
CDS	2657	1836	1313	731	416	2855	2221	1452	766	222	7885	4754	2952
5′UTR	1106	613	617	258	132	908	518	580	191	90	2238	1221	861
3′UTR	1135	785	381	369	233	698	512	307	178	140	1769	910	695
Other UTR	37	26	13	5	6	0	0	1	0	0	3	2	3
Total nucs.	38.7M	27.1M	9.6M	17.8M	10.4M	83.8M	54.7M	61.3M	63.2M	55.4M	130.0M	96.4M	75.3M
Total regs.	76,805	36,277	32,557	20,344	23,198	120,968	75,470	35,528	29,275	32,314	150,966	64,438	46,339

**Fig. 3:**
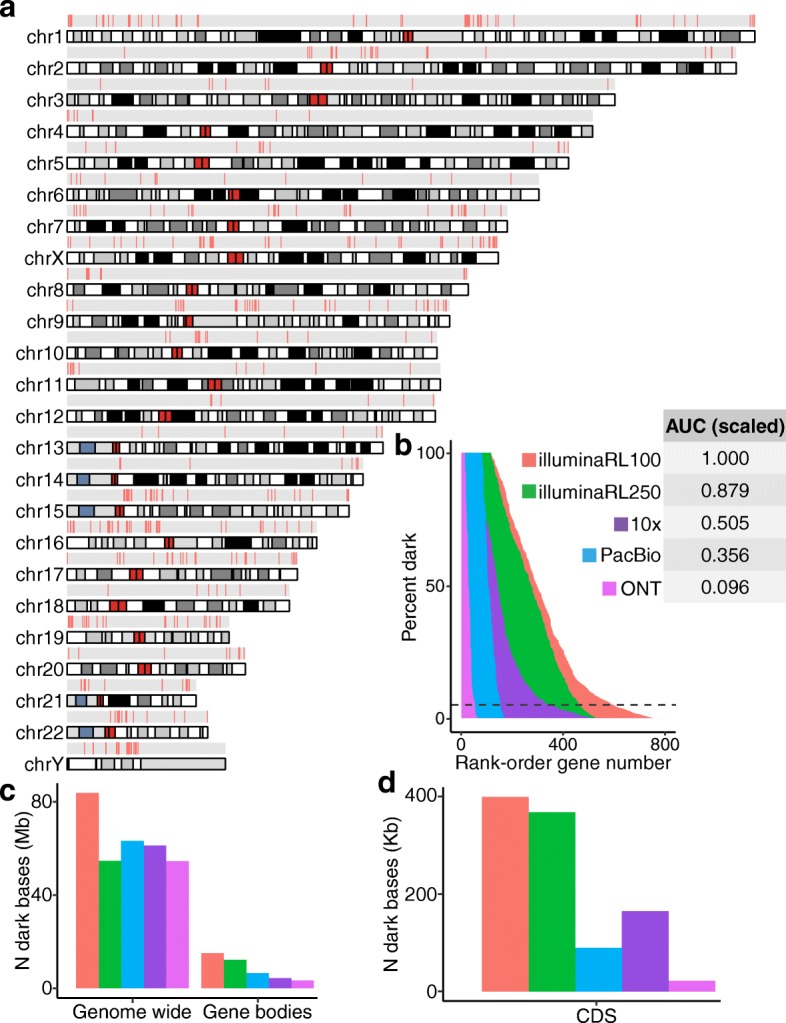
Dark coding regions occur throughout the genome and are largely resolved with long-read sequencing technologies. We identified 2855 dark coding (CDS) regions in 748 protein-coding genes that were dark by either depth or mapping quality (Additional file 2: Table S1; Additional file 3: Table S2). We identified 117 (15.6%) of the 748 protein-coding genes were 100% dark in CDS regions, 402 (53.7%) were at least 25% dark in CDS regions, and 592 (79.1%) were at least 5% dark in CDS regions (Additional file 2: Table S1). **a** We mapped all protein-coding gene bodies with a dark coding exon to the genome to visualize their genomic location and are generally spread throughout. There are several tight clusters of dark CDS regions on chromosomes 1, 9, 10, and Y, however. **b** We assessed how well increasing read lengths would resolve dark regions by assessing samples sequenced with Illumina whole-genome sequencing using 250-nucleotide read lengths, as well as long-read technologies 10x Genomics, Oxford Nanopore Technologies (ONT), and Pacific Biosciences (PacBio). Data from the samples sequenced using 250-nucleotide Illumina read lengths reduced the area under the curve (AUC) by 12.1% in CDS regions. Comparing long-read sequencing technologies to the standard Illumina 100-nucleotide read lengths, 10x Genomics, PacBio, and ONT reduced the area under the curve for CDS regions by approximately 49.5%, 64.4%, and 90.4%, respectively. The AUC for each technology is scaled in reference to Illumina sequencing based on 100-nucleotide read lengths (i.e., AUC for Illumina 100-nucleotide read lengths = 1). In contrast to overall results, PacBio outperformed 10x Genomics when looking only at CDS regions (see text). Most analyses focused on genes where at least 5% of the CDS nucleotides are dark, indicated by the dashed line. **c**, **d** We also calculated the raw number of dark nucleotides for each technology in GRCh38, genome wide, in full gene bodies, and in CDS regions

Focusing only on CDS regions, we identified 2855 dark CDS regions (> 460,000 nucleotides) across 748 protein-coding genes that were dark by either depth or mapping quality (Fig. 3a; Additional file 2: Table S1; Additional file 3: Table S2). We identified 117 (15.6%) of the 748 protein-coding genes that were 100% dark in CDS regions, 402 (53.7%) were at least 25% dark in CDS regions, and 592 (79.1%) were at least 5% dark in CDS regions (Fig. 3b; Additional file 2: Table S1).

### Most dark protein-coding regions are specifically camouflaged

Regions may be dark because of either low depth or low mapping quality, but the majority of regions are dark because of mapping quality, and many specifically because they are camouflaged (low mapping quality because of a duplication). We found that 3782 (62.5%) of the 6054 dark gene bodies are dark because of mapping quality, where 2716 (44.9%) were, in fact, camouflaged. Likewise, 436 (73.6%) of the 592 genes that were ≥ 5% dark in CDS regions were dark because they were camouflaged. We also measured the number of times each gene region was duplicated and found that 71.1% of gene regions were replicated three or fewer times in the genome, but 42 regions were duplicated ≥ 100 times (Additional file 1: Figure S2a), with the most repeated regions (six separate intronic regions totaling 833 nucleotides from *FGF12* intron six) being replicated 530 times in aggregate. Limiting to only CDS regions, we estimate that 76.2% are replicated three or fewer times, with 45 replicated ≥ 10 times (Additional file 1: Figure S2b), and the most repeated region was from *NBPF20*, in which 109 nucleotides were replicated 32 times.

### Linked- and long-read sequencing technologies resolve substantial portions of the dark regions

Data from the samples sequenced using 250-nucleotide Illumina read lengths reduced the percentage of dark nucleotides by 34.7%, 19.2%, and 8.1% genome-wide, for all gene bodies, and for only CDS regions, respectively, leaving 65.3%, 80.8%, and 91.9% of the original dark nucleotides, respectively (Fig. 3c, d; Additional file 4: Table S3; Additional file 5: Table S4). Comparing linked- and long-read sequencing technologies to the standard Illumina 100-nucleotide read lengths, the ONT platform performed best, both when assessing entire gene bodies, and when considering only CDS regions. Specifically, approximately 42.8%, 28.7%, and 22.1% of the nucleotides remained dark for all gene bodies for PacBio, 10x Genomics, and ONT, respectively (Fig. 3c; Additional file 6: Table S5; Additional file 7: Table S6; Additional file 8: Table S7; Additional file 9: Table S8; Additional file 10: Table S9; Additional file 11: Table S10). Similarly, approximately 22.3%, 41.2%, and 5.4% of CDS nucleotides remained dark for PacBio, 10x Genomics, and ONT, respectively (Fig. 3d; Additional file 6: Table S5; Additional file 7: Table S6; Additional file 8: Table S7; Additional file 9: Table S8; Additional file 10: Table S9; Additional file 11: Table S10). We also calculated the area under the curve (AUC) for each technology, where the AUC is based on the percentage of each gene that is dark. Compared to the AUC for 100-nucleotide Illumina read lengths, Illumina-250 read lengths, PacBio, 10x Genomics, and ONT resolved 12.1%, 64.4%, 49.5%, and 90.4% of CDS gene regions, respectively (Fig. 3b). Only 15 of 117 genes that were originally 100% dark remained 100% dark in the ONT data. In contrast to overall gene-body results, PacBio outperformed 10x Genomics when looking only at CDS regions (Fig. 3c, d). The long-read technologies improved over Illumina mostly by reducing the percentage of nucleotides that are dark by mapping quality (Additional file 1: Figure S1c). Surprisingly, the percentage of gene-body regions that are dark because of low depth is higher for long-read technologies than it is for Illumina (Additional file 1: Figure S1c).

### Important pathways and gene families are affected by dark and camouflaged regions

**Fig. 4:**
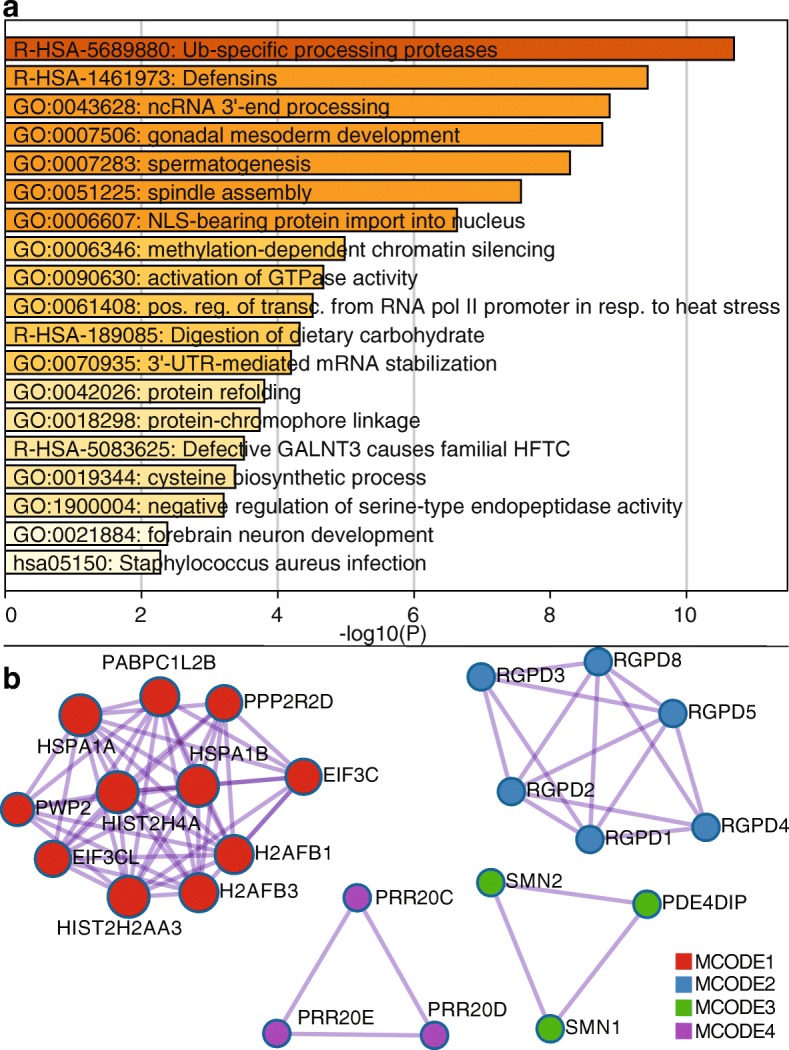
Pathways relevant to human health, development, and reproductive function are affected by dark and camouflaged genes. We characterized the pathways for dark and camouflaged genes using Metascape.org, including only genes where at least 5% of the CDS regions were dark (565 unique gene symbols; based on standard Illumina 100 nucleotide read lengths). **a** Specific pathway groups included Ub-specific processing proteases (R-HSA-5689880; logP = − 10.70), defensins (R-HSA-1461973; logP = − 9.43), ncRNA 3′-end processing (GO:0043628; logP = − 8.87), gonadal mesoderm development (GO:0007506; logP = − 8.76), spermatogenesis (GO:0007283; logP = − 8.29), spindle assembly (GO:0051225; logP = − 7.56), NLS-bearing protein import into nucleus (GO:0006607; logP = − 6.63), methylation-dependent chromatin silencing (GO:0006346; logP = − 4.98), activation of GTPase activity (GO:0090630; logP = − 4.67), and others. **b** Looking specifically at known protein-protein interactions, we found 103 proteins with 172 known interactions (Additional file 1: Figure S3) and, within those, identified four groups enriched for protein-protein interactions using the MCODE algorithm [28] (Fig. 4b). All four MCODE groups combined are primarily associated with RNA transport (hsa030313; logP = − 18.59; Additional file 1: Figure S4; accessed March 2019). Individually, the first group (MCODE1) is enriched for proteins involved in systemic lupus erythematosus (hsa05322; logP = − 6.55), cellular response to stress (R-HSA-2262752; logP = − 6.13), and RNA transport (hsa03013; logP = − 4.26; Additional file 1: Figure S5). The second group (MCODE2) is enriched with proteins involved in NLS-bearing protein import into nucleus (GO:0006607; logP = − 18.44; Additional file 1: Figure S6). The third and fourth groups do not have significant enrichment associations, likely because little is known about them; five of the six genes (*PRR20C*, *PRR20D*, *PRR20E*, *SMN1*, and *SMN2*) are completely or nearly 100% camouflaged, and several do not even have known expression measurements in GTEx [29] (Additional file 1: Figures S7-S9)

Because such a large number of genes are dark, we characterized the pathways for genes that are not fully represented in standard Illumina short-read sequencing (100-nucleotide reads) datasets. We included all genes where at least 5% of the CDS regions were dark (565 unique gene symbols) and identified several pathways that are important in human health, development, and reproductive function (Fig. 4a; Additional file 12: Table S11). Specific pathway groups included Ub-specific processing proteases (R-HSA-5689880; logP = − 10.70), defensins (R-HSA-1461973; logP = − 9.43), ncRNA 3′-end processing (GO:0043628; logP = − 8.87), gonadal mesoderm development (GO:0007506; logP = − 8.76), spermatogenesis (GO:0007283; logP = − 8.29), spindle assembly (GO:0051225; logP = − 7.56), NLS-bearing protein import into nucleus (GO:0006607; logP = − 6.63), methylation-dependent chromatin silencing (GO:0006346; logP = − 4.98), activation of GTPase activity (GO:0090630; logP = − 4.67), and others. Some specific gene families involved in these pathways include 21 ubiquitin-specific 17-like family members (e.g., USP17L3), 12 defensin genes (e.g., DEFA1 and DEFB4A), 6 testis-specific proteins (e.g., TSPY2), and 13 golgin genes (e.g., GOLGA6B; Additional file 12: Table S11).

Looking specifically at known protein-protein interactions, we found 103 proteins with 172 known interactions (Additional file 1: Figure S3) and, within those, identified four groups enriched for protein-protein interactions using the MCODE algorithm [28] (Fig. 4b). All four MCODE groups combined are primarily associated with RNA transport (hsa030313; logP = − 18.59; Additional file 1: Figure S4; accessed March 2019). Individually, the first group (MCODE1) is enriched for proteins involved in systemic lupus erythematosus (hsa05322; logP = − 6.55), cellular response to stress (R-HSA-2262752; logP = − 6.13), and RNA transport (hsa03013; logP = − 4.26; Additional file 1: Figure S5). The second group (MCODE2) is enriched with proteins involved in NLS-bearing protein import into nucleus (GO:0006607; logP = − 18.44; Additional file 1: Figure S6). The third and fourth groups do not have significant enrichment associations, likely because little is known about them; five of the six genes (*PRR20C*, *PRR20D*, *PRR20E*, *SMN1*, and *SMN2*) are completely or nearly 100% camouflaged, and several do not even have known expression measurements in GTEx [29] (Additional file 1: Figures S7-S9).

### There are 76 dark genes with known mutations associated with 326 human diseases

**Fig. 5:**
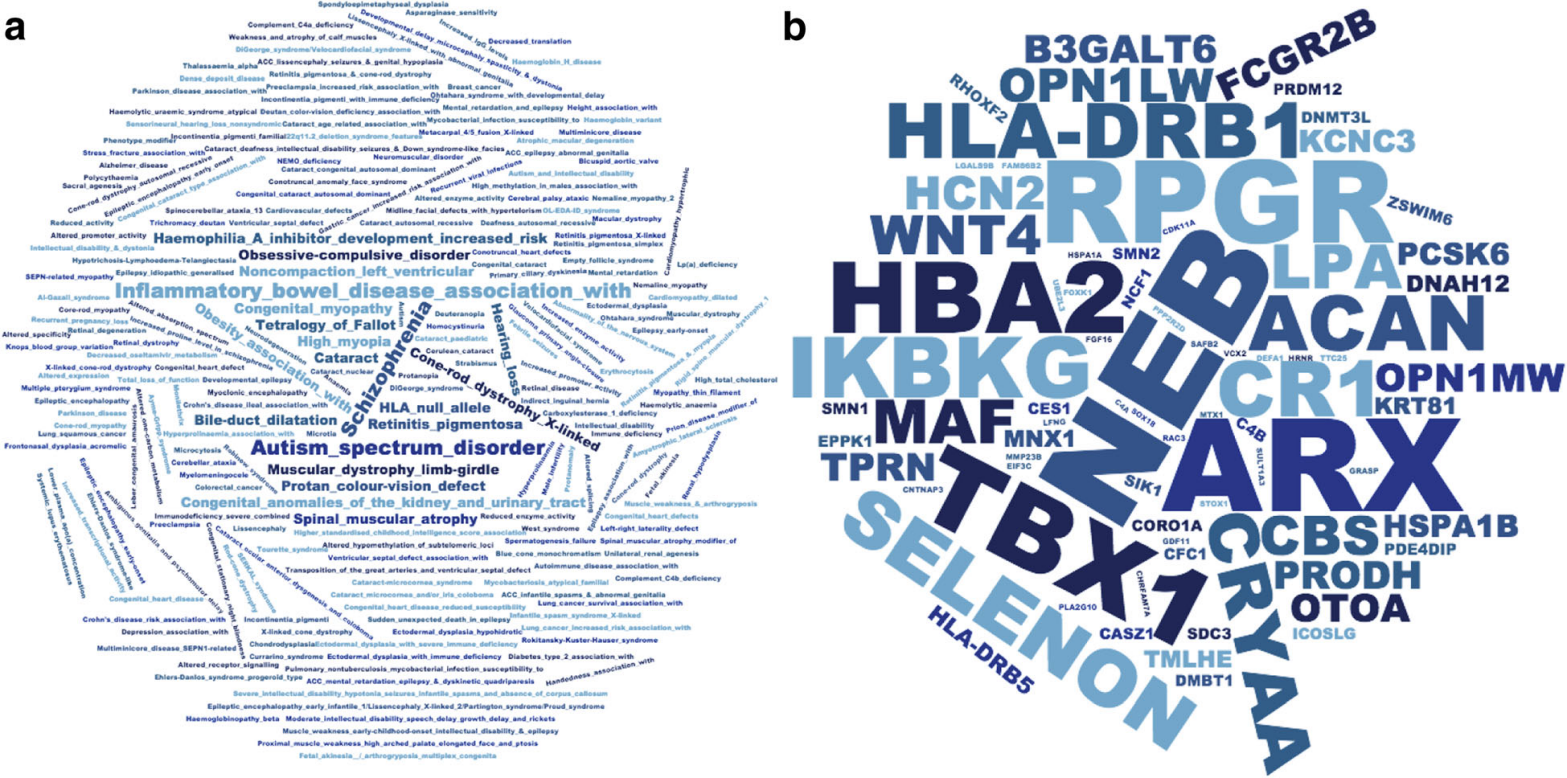
Seventy-six dark genes (≥ 5% CDS) are associated with 326 human diseases, including autism, inflammatory bowel disease, and others. We found 76 genes ≥ 5% dark CDS that harbor mutations associated with 326 unique human diseases, according to the Human Gene Mutation Database (HGMD). **a** Some of the diseases with the most known associated genes include autism spectrum disorder, schizophrenia, hearing loss, spinal muscular atrophy, and inflammatory bowel disease. Word size represents the number of genes associated with each disease. These data demonstrate the number of diseases impacted by genes that are at least 5% dark CDS, and how important it is to completely resolve dark regions. We also performed an enrichment analysis, where the diseases most enriched for dark genes included color blindness (protan color vision defect), X-linked cone-rod dystrophy, and spinal muscular atrophy (Additional file 1: Figure S10). **b** Similarly, we quantified the number of diseases each gene was associated with and identified many disease-relevant genes with large portions of dark CDS regions that may harbor critical disease-modifying mutations that currently go undetected. Some of the genes with the most known disease associations include *ARX* (12.8% dark CDS), *NEB* (9.5% dark CDS), *TBX1* (10.6% dark CDS), *RPGR* (8.6% dark CDS), *HBA2* (9.5% dark CDS), and *CR1* (26.0% dark CDS). *CR1* is particularly notable for neuroscientists and Alzheimer’s disease geneticists, patients, and their caregivers, given that CR1 is a top-ten Alzheimer’s disease gene. Other notable genes include SMN1 (94.6% dark CDS) and SMN2 (88.0% dark CDS), which are known to harbor mutations (in camouflaged regions) that are involved in spinal muscular atrophy (SMA) and are implicated in ALS. HSPA1A (53.0% dark CDS) and HSPA1B (51.5% dark CDS) also encode two primary 70-kilodalton (kDa) heat-shock proteins. Heat-shock proteins have been implicated in ALS [31, 32]

To assess the potential impact missing mutations in dark genes may have on human disease genetics, we measured the number of dark genes with at least 5% dark CDS that have mutations known to be involved in human disease; we calculated the number of genes that are ≥ 5% dark CDS with a mutation in the Human Gene Mutation Database (HGMD) [30]. We found 76 genes associated with 326 unique human diseases (Fig. 5a). Some of the diseases with the most known associated genes include autism spectrum disorder, schizophrenia, hearing loss, spinal muscular atrophy, and inflammatory bowel disease. Some of the diseases most represented in our data are not surprising, given the number of genes involved in the disease, but these data demonstrate the number of diseases impacted by genes that are at least 5% dark CDS. We also performed an enrichment analysis, where the diseases most enriched for dark genes included color blindness (protan color vision defect), X-linked cone-rod dystrophy, and spinal muscular atrophy (Additional file 1: Figure S10).

Similarly, we quantified the number of diseases each gene was associated with (Fig. 5b). We identified many disease-relevant genes with large portions of dark CDS regions that may harbor critical disease-modifying mutations that currently go undetected. Some of the genes with the most known disease associations include *ARX* (12.8% dark CDS), *NEB* (9.5% dark CDS), *TBX1* (10.6% dark CDS), *RPGR* (8.6% dark CDS), *HBA2* (9.5% dark CDS), and *CR1* (26.0% dark CDS). The *CR1* gene is particularly notable given that *CR1* is a top-ten Alzheimer’s disease gene. Other notable genes include *SMN1* (94.6% dark CDS) and *SMN2* (88.0% dark CDS), which are known to be involved in spinal muscular atrophy (SMA) and ALS [33–35]. *HSPA1A* (53.0% dark CDS) and *HSPA1B* (51.5% dark CDS) also encode two primary 70-kilodalton (kDa) heat-shock proteins, a family of proteins that have been implicated in ALS [31, 32].

### Camouflaged genes are consistently dark in gnomAD, but dark-by-depth genes may be sample or dataset specific

**Fig. 6:**
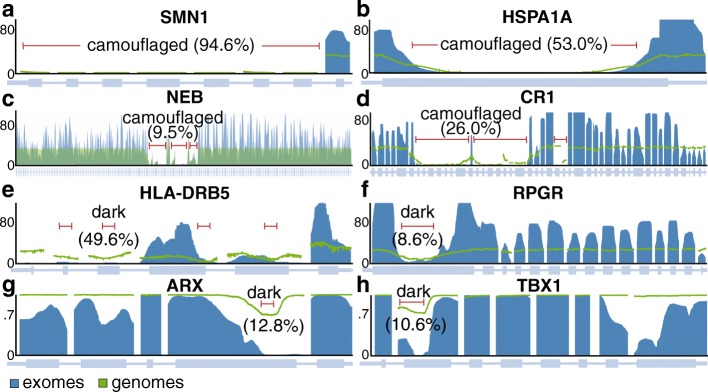
Camouflaged genes are consistently dark in gnomAD, but dark-by-depth genes may be sample or dataset specific. Many dark genes are specifically camouflaged (Additional file 13: Table S12; Additional file 14: Table S13), but many are dark by depth; we found that camouflaged regions in the ADSP are consistently dark in the gnomAD consortium data (http://gnomad.broadinstitute.org/) [36]. Dark-by-depth regions may be more variable between samples and datasets, however, suggesting these regions may be sensitive to specific aspects of whole-genome sequencing (e.g., library preparation) or downstream analyses. **a** *SMN1* and *SMN2* are camouflaged by each other (only *SMN1* shown). Both genes contribute to spinal muscular atrophy and have been implicated in ALS. **b** *HSPA1A* and *HSPA1B* are also camouflaged by each other (only *HSPA1A* shown). The heat-shock protein family has been implicated in ALS. **c** *NEB* (9.5% dark CDS) is a special case that is camouflaged by itself. *NEB* is associated with 24 diseases in the HGMD, including nemaline myopathy, a hereditary neuromuscular disorder. *NEB* is a large gene; thus, 9.5% dark CDS translates to 2424 protein-coding bases. **d** *CR1* is a top Alzheimer’s disease gene that plays a critical role in the complement cascade as a receptor for the C3b and C4b complement components, and potentially helps clear amyloid-beta (Aβ) [37–39]. *CR1* is also camouflaged by itself, where the repeated region includes the extracellular C3b and C4b binding domain. The number of repeats and density of certain isoforms have been associated with Alzheimer’s disease [21, 40–43]. **e** *HLA-DRB5* is dark by depth in the ADSP and gnomAD data. *HLA-DRB5* has been implicated in several diseases, including Alzheimer’s disease. **f** *RPGR* is likewise dark in ADSP and gnomAD and is associated with several eye diseases, including retinitis pigmentosa and cone-rod dystrophy. **g** *ARX* is dark-by-depth, but varies by sample or cohort, as approximately 70% of gnomAD samples are not strictly dark by depth. *ARX* is associated with diseases including early infantile epileptic encephalopathy 1 (EIEE1) and Partington syndrome. **h** Similarly, *TBX1* is not strictly dark by depth in approximately 70% of gnomAD samples. The *Y* axes for figures **a**–**f** indicate median coverage in gnomAD (blue = exomes; green = genomes), whereas the *Y* axes in **g**, **h** represent the proportion of gnomAD samples that have > 5x coverage. Dark and camouflaged regions, as well as the percentage of each gene’s CDS region that is dark, are indicated by red lines. Dark regions in exome data are either similar or more pronounced than what we observed in whole-genome data, highlighting that dark and camouflaged regions are generally magnified in whole-exome data. For interest, we also discovered that *APOE*—the top genetic risk for Alzheimer’s disease [44–46]—is approximately 6% dark CDS (by depth) for certain ADSP samples with whole-genome sequencing, and the same region is dark in gnomAD whole-exome data (Additional file 1: Figure S11)

Although many dark genes are specifically camouflaged (Additional file 13: Table S12; Additional file 14: Table S13), many are dark by depth in the ADSP data; upon manual comparison between whole-genome sequencing data from the ten ADSP males and coverage plots from the gnomAD consortium dataset (http://gnomad.broadinstitute.org/) [36], we found that camouflaged regions in the ADSP males are consistently dark in the gnomAD data, demonstrating that these camouflaged regions are consistent across datasets. The ADSP data are also included in gnomAD, but they only make up approximately 15% of the data. The dark-by-depth regions are more variable between samples and datasets, however, suggesting these regions may be sensitive to specific aspects of whole-genome sequencing (e.g., library preparation) or downstream analyses. Specific camouflaged genes include *SMN1* and *SMN2* (Fig. 6a), *HSPA1A* and *HSPA1B* (Fig. 6b), *NEB* (9. Fig. 6c), and *CR1* (Fig. 6d). Specific dark-by-depth genes include *HLA-DRB5* (Fig. 6e), *RPGR* (Fig. 6f), *ARX* (Fig. 6g), and *TBX1* (Fig. 6h). All four camouflaged genes are also dark in the gnomAD data. A manual inspection of our dark-by-depth gene list, however, suggests most are not completely dark in gnomAD, but vary by sample or dataset. Specifically, *HLA-DRB5* and *RPGR* in gnomAD appear to be consistent with the ADSP data; *ARX* and *TBX1*, however, only appear to be dark in a portion of the gnomAD samples, where about 30% of samples have ≤ 5 reads in their respectively defined dark regions. Dark regions are either similar or more pronounced in the gnomAD whole-exome data than what we observed in the whole-genome data (Figs. 6a–h), highlighting that dark and camouflaged regions are generally magnified in whole-exome data; this is likely because of differences in library preparation and shorter read lengths in exome data. For interest, we also found that *APOE*—the top genetic risk for Alzheimer’s disease [44–46]—is approximately 6% dark CDS (by depth) for certain ADSP samples with whole-genome sequencing, and the same region is dark in gnomAD whole-exome data (Additional file 1: Figure S11). It is possible some of the dark regions we identified in standard short-read whole-genome data are specific to the ADSP samples, but additional work can clarify this issue. In either case, *dark-by-depth regions* (Additional file 15: Table S14; Additional file 16: Table S15) *should be interrogated within individual datasets, and perhaps for individual samples as a quality control measure*.

*SMN1* and *SMN2* are camouflaged by each other, where both genes are known to contribute to spinal muscular atrophy, and have been implicated in ALS. *HSPA1A* and *HSPA1B* are also camouflaged by each other, and the heat-shock protein family has been implicated in ALS [33, 35]. *NEB* is a special case that is camouflaged by itself (rather than another gene), and is associated with 24 diseases in the HGMD, including nemaline myopathy, a hereditary neuromuscular disorder. *NEB* is a large gene (249,151 nucleotides; 25,577 CDS nucleotides); thus, ~ 9.5% dark CDS translates to 2424 dark protein-coding bases. *CR1* is a top Alzheimer’s disease gene that plays a critical role in the complement cascade as a receptor for the C3b and C4b complement components, and potentially helps clear amyloid-beta (Aβ) [37–39]. Like *NEB*, *CR1* is also camouflaged by itself, where the repeated region actually includes the extracellular C3b and C4b binding domain. The number of repeats and density of certain isoforms have been associated with Alzheimer’s disease [21, 40–43].

We found *HLA-DRB5* is dark by depth in the ADSP and gnomAD data and has been implicated in several diseases, including Alzheimer’s disease. *RPGR* is likewise dark in ADSP and gnomAD and is associated with several eye diseases, including retinitis pigmentosa and cone-rod dystrophy. We identified *ARX* as a dark-by-depth gene, but this gene appears to vary by sample or cohort, as only approximately 30% of gnomAD samples are strictly dark by depth, using our cutoff of ≤ 5 reads. *ARX* is associated with diseases including early infantile epileptic encephalopathy 1 (EIEE1) [47] and Partington syndrome [48]. Similarly, *TBX1*, which harbors mutations that cause the same phenotype as 22q11.2 deletion syndrome [49], is dark by depth in only approximately 30% of gnomAD samples.

### Linked- and long-read technologies resolve many camouflaged regions, with variable success

**Fig. 7:**
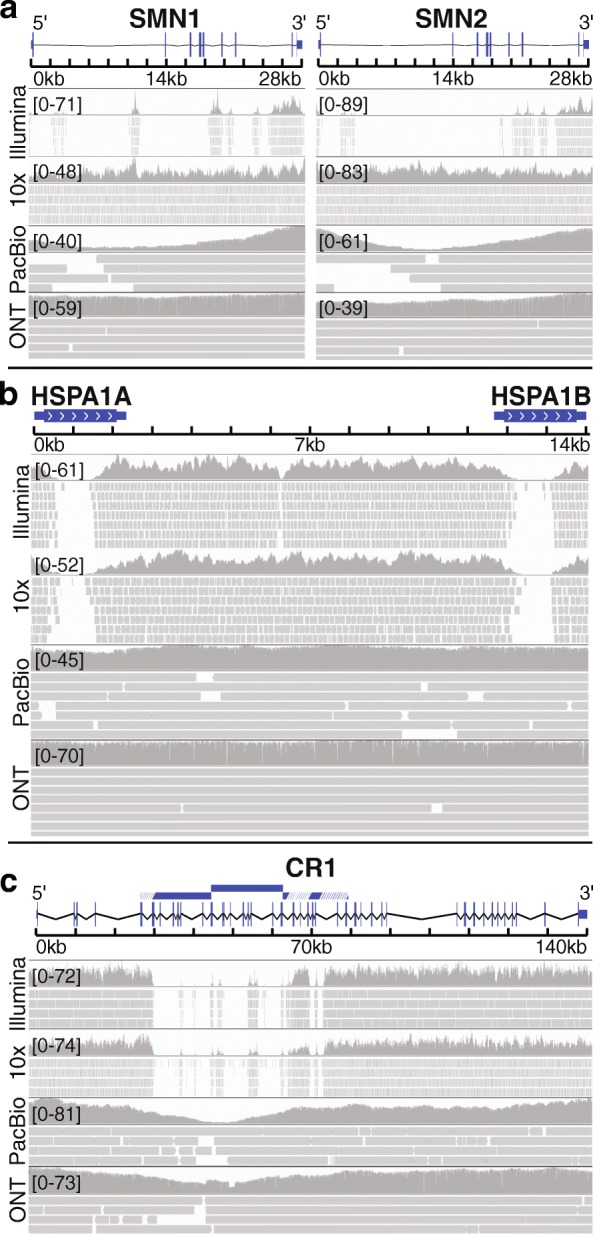
Long-read technologies resolve many camouflaged regions, with variable success. We found that ONT’s long-read technology appeared to resolve all camouflaged regions well with the high sequencing depth. PacBio performed similarly well, and 10x Genomics performs well under certain circumstances. **a** *SMN1* and *SMN2* were 94.6% and 88.0% camouflaged CDS, respectively, based on standard Illumina sequencing with 100-nucleotide read lengths (illuminaRL100). Both genes were 0% camouflaged CDS for 10x Genomics, PacBio, and ONT data. 10x Genomics and ONT perform particularly well in these genes, with consistently high mapping coverage. **b** *HSPA1A* and *HSPA1B* were 53.0% and 51.5% camouflaged CDS, respectively, based on illuminaRL100 data. Both genes were 0% camouflaged CDS based on ONT and PacBio data and were 45.8% and 51.8% camouflaged CDS based on 10x Genomics data. In contrast to the results for *SMN1* and *SMN2*, 10x Genomics was unable to resolve the *HSPA1A* and *HSPA1B* camouflaged regions. **c** *CR1* was 26.0% camouflaged CDS based on illuminaRL100. 10x Genomics did not improve coverage for *CR1*; the region remained 26.4% camouflaged CDS. Both ONT and PacBio were 0% camouflaged CDS. While both PacBio and ONT were able to fill the camouflaged region, coverage dropped throughout the region, particularly for PacBio. The duplicated region is indicated by blue bars, where white lines indicate regions that have diverged sufficiently for short-reads to align uniquely. Regions were visualized with IGV. Reads with a MAPQ < 10 were filtered, and insertions, deletions, and mismatches are not shown

We selected three camouflaged gene regions to highlight common strengths and differences for how well each linked- or long-read sequencing technology addresses the camouflaged region, including *SMN1* and *SMN2* (Fig. 7a), *HSPA1A* and *HSPA1B* (Fig. 7b), and *CR1* (Fig. 7c). The *SMN1* and *SMN2* genes are camouflaged by each other (gene duplication), as are *HSPA1A* and *HSPA1B*. *CR1*, however, is a special case, where it is camouflaged by a repeated region within itself. Only ONT appeared to be capable of fully addressing the camouflaged region for all three genes. 10x Genomics also performed well under certain circumstances, such as *SMN1* and *SMN2* (regions where the duplication is > 50 kb away), but did not perform well for *HSPA1A* and *HSPA1B*. PacBio performed well for *HSPA1A/HSPA1B*, but did not perform as well as ONT in *CR1* and the *SMN1/SMN2* region.

### Many camouflaged regions can be rescued, including in standard short-read sequencing data

There are many large-scale whole-genome or whole-exome sequencing projects across tens of thousands of individuals that are either completed or underway for a variety of diseases, including cancer (e.g., The Cancer Genome Atlas (TCGA)), autism spectrum disorder (e.g., The Autism Sequencing Consortium (ASC)), Alzheimer’s disease (e.g., The Alzheimer’s Disease Sequencing Project (ADSP)), Parkinson’s disease (e.g., The Parkinson’s Progression Markers Initiative (PPMI)), and ALS (e.g., Target ALS and CReATe). All of these datasets are affected by dark and camouflaged regions that may harbor mutations that either are driving or modify disease in patients. Ideally, all samples would be re-sequenced using the latest technologies over time, but financial resources and biological samples are limited, making it essential to maximize the utility of existing data.

**Fig. 8:**
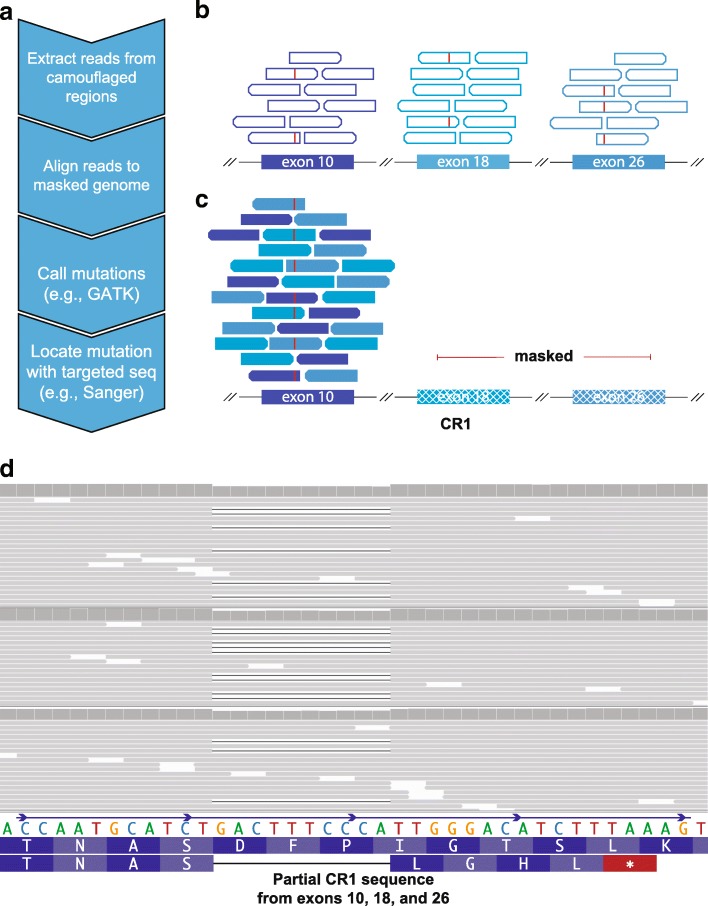
Many camouflaged regions can be rescued, including *CR1*, even in standard short-read sequencing data. Many large-scale whole-genome or whole-exome sequencing projects exist, covering tens of thousands of individuals. All of these datasets are affected by dark and camouflaged regions that may harbor mutations that either drive or modify disease in patients. Ideally, all samples would be re-sequenced using the latest technologies over time, but financial resources and biological samples are limited, making it essential to maximize the utility of existing data. We developed a method to rescue mutations in most camouflaged regions, including for standard short-read sequencing data. When confronted with a sequencing read that aligns to two or more regions equally well (with high confidence), most aligners (e.g., BWA [11–13]) will randomly assign the read to one of the regions with a low mapping quality (e.g., MAPQ = 0 for BWA). **a** Because the reads are already aligned to one of the regions, we can use the following steps to rescue mutations in most camouflaged regions: (1) extract reads from camouflaged regions, (2) mask all highly similar regions in the reference genome, except one, and re-align the extracted reads, (3) call mutations using standard methods (adjusting for ploidy), and (4) determine precise location using targeted sequencing (e.g., long-range PCR combined with Sanger, or targeted long-read sequencing [1]). Without competing camouflaged regions to confuse the aligner, the aligner will assign a high mapping quality, allowing variant callers to behave normally. **b** Exons 10, 18, and 26 in *CR1* are identical, according to the reference genome. Standard aligners will randomly scatter reads matching that sequence across these exons and assign a low mapping quality (e.g., MAPQ = 0 for BWA; indicated as hollow reads). Red lines indicate an individual’s mutation that exists in one of these exons, but reads containing this mutation also get scattered and assigned a low mapping quality. **c** By masking exons 18 and 26, we can align all of these reads to exon 10 with high mapping qualities to determine whether a mutation exists. We cannot determine at this stage which of the three exons the mutation is actually located in, but researchers can test association with a given disease to determine whether the mutation is worth further investigation. **d** As a proof of principle, we rescued approximately 4214 exonic variants in the ADSP (TiTv = 2.26) using our method, including a frameshift mutation in *CR1* (MAF = 0.00019) that is found in five cases and zero controls (three representative samples shown). The frameshift results in a stop codon shortly downstream. The ADSP is not large enough to formally assess association between the *CR1* frameshift and Alzheimer’s disease, but we believe the mutation merits follow-up studies given its location (*CR1* binding domain) and *CR1*’s strong association with disease

Using a strategy similar to that proposed by Robert and Watson [10], we have developed a method to rescue mutations in most camouflaged regions, including for standard Illumina short-read sequencing data. When confronted with a sequencing read that aligns to two or more regions equally well (with high confidence), most aligners (e.g., BWA [11–13]) will randomly map the read to one of the regions and assign a low mapping quality (MAPQ = 0 for BWA, or MAPQ = 1 for novoalign). Because the reads are already aligned to one of the regions, we can use the following steps to rescue mutations in most camouflaged regions (Fig. 8): (1) extract reads from camouflaged regions; (2) mask all highly similar regions in the reference genome, except one, and re-align the extracted reads; and (3) call mutations using standard methods, while accounting for increased ploidy and potential reference-based artifacts. Reference-based artifacts arise when regions within a given camouflaged set are not 100% identical, causing false positives when reads from both regions are aligned to a single region. Without competing camouflaged regions to confuse the aligner, the aligner will assign a high mapping quality, allowing variant callers to behave normally. This will enable researchers to identify mutations that exist in one of the camouflaged regions, but cannot indicate which specific region the mutation originated from (Fig. 8). After rescuing these mutations, researchers can then perform association studies to determine whether any of the mutations may be implicated in disease, and follow up with targeted sequencing methods to determine the exact camouflage region a mutation lies in.

### Re-alignment rescues approximately 4214 exonic variants, including a rare ten-nucleotide frameshift deletion in *CR1*

As a proof of principle, we applied our method to the Alzheimer’s Disease Sequencing Project (ADSP) case-control data [50] to approximate the number of potential mutations our approach could rescue. The ADSP is a large sequencing project organized, in part, to identify functional mutations that influence Alzheimer’s disease development. Across 10,933 samples from the ADSP, excluding all reference-based artifacts, and variants with a quality by depth (QD) ≤ 2, we were able to rescue approximately 4214 exonic variants with a transition-transversion ration (Ti/Tv) of 2.26 from 137 camouflaged region sets, that are spread across 748 camouflaged genes (Additional file 1: Figure S12; VCF will be provided to the ADSP). Using a more stringent QD (excluding variants with QD ≤ 3), we rescued 3343 variants with a Ti/Tv ratio of 2.35. We only included camouflaged regions from CDS exons for all genes, including those that are < 5% dark CDS.

Because *CR1* is a top-10 Alzheimer’s disease gene, we then specifically interrogated it using our method (Fig. 8) for any functional mutations that could be involved in Alzheimer’s disease. Using 13,142 ADSP samples, we identified a rare ten-nucleotide frameshift deletion that is found in five cases but zero controls, all of which are heterozygous (Fig. 8d). Three of the five carriers were of European descent and two were Caribbean Hispanic. All five samples were from the ADSP case-control series; thus, we did not expect them to be related. We verified the three European carriers were unrelated (KING-robust kinship < 0.0442) [51], but the two Caribbean Hispanic carriers appear to be first-degree relatives (KING-robust kinship = 0.3356). For interest, only one of the individuals carried a single *APOE*ε4 allele (ε3/ε4). The other four individuals were homozygous for *APOE*ε3 (ε3/ε3). We were able to determine that the frameshift deletion is in one of exons 10, 18, or 26. We estimate a cohort of approximately 70,000 cases and controls would have approximately 80% statistical power to formally assess this mutation’s involvement in Alzheimer’s disease, assuming a relative risk (RR) of 3.3, at an alpha of 0.0001.

## Discussion

While researchers have known for years that dark regions exist in standard short-read sequencing data, little work has been done to characterize the breadth of the issue and to develop possible solutions until more financially feasible linked- or long-read sequencing options are available. Short-read sequencing is unable to adequately address camouflaged regions because the reads cannot fully span camouflaged regions to properly align homologous nucleotides. Linked- and long-read sequencing technologies, such as those from 10x Genomics, Oxford Nanopore Technologies (ONT), and Pacific Biosciences (PacBio), have the potential to address many camouflaged regions because these technologies have median read lengths measured in thousands of nucleotides, rather than only 100–300 nucleotides from standard short-read sequencing technologies (e.g., Illumina). Recent work has even demonstrated that mappable ONT reads can exceed two million nucleotides (e.g, 2,272,580) [52, 53], showing future potential for addressing large camouflaged regions.

In this study, we systematically characterized dark and camouflaged gene regions and proposed a method to address most camouflaged regions in long- or short-read sequencing data. Our solution is specifically applicable to camouflaged regions, not regions that are dark by depth, simply because there are no reads available in those regions. While our solution is conceptually simple, implementing the solution systematically was challenging because of many intricate details, including increased zygosity, and would ideally be integrated into the original alignment and variant-calling process. While the original implementation was challenging, we provide the resulting .bed files for both GRCh37 and GRCh38 that are necessary to rescue mutations from camouflaged regions in any human re-sequencing dataset (https://github.com/mebbert/Dark_and_Camouflaged_genes). We also provide all of our data and source code. The .bed files and source code should make implementing our method relatively straightforward for other groups. As a proof of concept, we were able to rescue approximately 4214 variants in the ADSP dataset from 137 sets of camouflaged gene regions, which are spread across 748 camouflaged genes. Included in these rescued mutations is a ten-nucleotide frameshift deletion in *CR1* found in five ADSP cases and zero controls.

The number of genes affected by dark and camouflaged regions was surprisingly high. We identified 36,794 total dark regions across 6054 gene bodies, 3804 of which were protein coding genes. We found that 27,982 of the dark regions were intronic and 2855 were in protein-coding exons (CDS). Others were in pseudogenes (1232) and lincRNAs (753). While most of the dark regions were non-coding (e.g., intronic), these regions may still harbor important mutations that drive or modify human diseases. For example, there are many examples of mutations in non-coding regions driving disease, including repeat expansions [1, 54–61], splice-site mutations (these may be intronic or exonic) [62–76], and regulatory mutations (e.g., UTR regions) [77–86]. There are also many lincRNAs associated with disease [87–96].

There are many patients with diseases known to be genetically inherited, yet remain genetically unexplained because the patients do not have any of the known mutations. Many of the genes we identified as being at least partially dark are known to be involved in numerous diseases, including Alzheimer’s disease, ALS, SMA, autism spectrum disorder, schizophrenia, and others; functional mutations that modify disease likely lie in some of these dark and camouflaged regions. For example, *SMN1* and *SMN2* are mostly dark (camouflaged) and are known to harbor mutations that cause disease [62, 64–66]. *CR1* is another dark gene that is 26.5% dark CDS, being camouflaged to itself, and is strongly implicated in Alzheimer’s disease. In fact, the *CR1* camouflaged region includes the C3b and C4b protein binding sites, repeated several times. Interestingly, the *C4B* gene (encodes the C4b protein) is also 72.8% dark CDS (camouflaged) and may be involved in disease [97, 98]. We are confident that rescuing mutations from camouflaged regions will have a meaningful impact on disease research and may explain some of the missing heritability of Alzheimer’s disease [18, 99–101] and other diseases.

A large number of gene bodies (527) were 100% dark, which means they are entirely overlooked in standard whole-exome, whole-genome, and RNA sequencing studies [10]. Additionally, more than 1600 gene bodies, or nearly 25%, were at least 25% dark and more than 2100 (35.2%) were at least 5% dark; of these, 748 protein-coding genes were at least 5% dark within CDS regions. Understanding what role these genes play in human health and disease will require being able to resolve them in DNA and RNA sequencing experiments.

A critical decision for future large-scale sequencing projects will be regarding which sequencing technology is ideal to maximize the probability of identifying functional mutations driving disease. Unfortunately, the answer is not clear, as each technology has its pros and cons. Based on our results, the ONT platform performed best, overall, resolving 77% of dark gene-body regions (Additional file 1: Figure S1b). Current costs may be prohibitive for large studies, however. The 10x Genomics platform resolved 64.3% of dark gene-body regions, when compared to standard Illumina sequencing. PacBio resolved 49.5% of dark gene-body regions. Even increasing Illumina read lengths from 100 to 250 made a sizeable difference, overall, resolving 12.2% of dark gene-body regions. Both the PacBio and ONT data used in this study had shorter median read lengths than expected, suggesting both technologies can likely perform better than our estimates.

Focusing only on CDS regions, there were 2855 dark CDS regions across 748 protein-coding genes, based on Illumina 100-nucleotide read lengths. ONT outperformed other long-read technologies, resolving 90.4% of dark CDS regions. PacBio and 10x Genomics resolved 64.4% and 49.5%, respectively. We found that 10x Genomics and ONT performed well in the *SMN1* and *SMN2* genes (Fig. 7), attaining consistently deep, high-quality coverage throughout. PacBio coverage declined in the interior regions of the genes. In other cases, such as *CR1* and *NEB*, 10x Genomics was unable to improve on standard Illumina sequencing, but ONT was able to largely resolve the region. PacBio also performed relatively well, but both ONT and PacBio required higher than normal sequencing depth for those technologies. We believe that 10x Genomics can correct the issues we observed in *CR1* and *NEB*, by implementing a more sophisticated version of our method that also incorporates evidence from their linked-read technology.

Whether each technology is able to reliably resolve dark and camouflaged regions is an important consideration for choosing the best sequencing technology, but we should also consider how reliably each technology is able to resolve structural mutations. In a previous study, we tested how well ONT and PacBio are able to traverse challenging repeat expansions, and whether they are amenable to genetic discovery [1]. We found that both technologies are well-suited, but we have not assessed performance of the 10x Genomics platform across long repeat expansions.

The primary challenge with ONT and PacBio long-read sequencing is, of course, the high error rate, which can be overcome through deeper sequencing because errors in ONT and PacBio sequencing are mostly random [102, 103]. Ultimately, we are confident that, as long-read error rates improve, and costs continue to decline, long-read technologies will be the preferred sequencing choice for large-scale sequencing projects, especially when considering structural mutations.

We identified dark and camouflaged regions in this study by averaging data across ten males with deep Illumina whole-genome sequencing, using 100-nucleotide read lengths. We assessed how well long-read sequencing technologies (PacBio, ONT, and 10X genomics) resolve these regions, but our measurements should only be considered estimates. While long-read sequencing technologies are becoming more common, we were unable to obtain more than one male individual for each long-read technology; we needed male samples to assess all chromosomes, including the Y chromosome. Additionally, the samples we used for each long-read technology were sequenced at a much higher depth than is currently typical for a re-sequencing effort, which is likely over estimating the number of dark regions they resolve for the average use case. Our measurements should be a reasonable estimate of reality, however, and future analyses will be able to refine our estimates.

We used whole-genome sequencing to assess dark and camouflaged regions, but this problem is magnified in whole-exome data, which many large-scale sequencing studies are based on, either completely, or in part. Whole-exome data are typically generated using even shorter read lengths. They are also generally based on capture, which means certain exons are not fully represented. *APOE* is a prime example, where it is typically well-covered in whole-genome data, but a portion is dark in whole-exome data (Additional file 1: Figure S11). With *APOE* harboring the largest genetic risk factors for Alzheimer’s disease, it is important to properly characterize the entire gene.

In this study, we characterized dark and camouflaged gene bodies and demonstrated several disease-relevant genes where a significant portion is dark in standard short-read sequencing data, including *SMN1* and *SMN2*, *CR1*, and sometimes even *APOE*. We also identified a rare ten-nucleotide frameshift deletion in *CR1* that is found in five ADSP cases and zero controls, as a proof of principle (Fig. 8d). Using our method (Fig. 8), we were able to determine that the frameshift deletion is in one of exons 10, 18, or 26. With *CR1* being a top Alzheimer’s disease gene without any known functional mutations, we believe it will be important to assess this mutation in a large cohort, to determine whether it plays a role in disease development and progression. We have also proposed a solution to address most camouflaged genes in sequencing data and believe that our approach has the potential to identify functional mutations that are influencing development across a range of diseases, but are currently entirely overlooked by standard short-read sequencing approaches.

## Conclusion

There remain thousands of potentially important genomic regions that are overlooked with short-read sequencing, but are largely resolved by linked- or long-read technologies. While these regions represent only a small portion of the entire genome or exome, many of these regions are known to be important in human health and disease. Equally important, however, is that the impact of many other genes is entirely unknown because they are 100% dark. We presented a method that can resolve most camouflaged regions that we believe will help researchers identify mutations that are involved in disease. As a proof of principle, we rescued approximately 4214 variants in the ADSP dataset, including a ten-nucleotide frameshift mutation in *CR1*. While we cannot formally assess the *CR1* frameshift mutation in Alzheimer’s disease (insufficient sample-size), we believe it is worth investigating in a larger cohort. In the long-term, we believe that linked- and long-read sequencing technologies will be the best solution for resolving dark and camouflaged regions.

## Methods

### Sample selection and preparation

To identify dark and camouflaged regions, and to assess how well other technologies address them, we selected samples from each technology and read length. All samples were aligned to GRCh37, GRCh38, and GRCh38+alt. To assess dark and camouflaged regions in standard Illumina sequencing with 100-nucleotide read lengths, we selected ten unrelated male control samples from the Alzheimer’s Disease Sequencing Project (ADSP) where deep whole-genome sequencing had been performed by randomly selecting one male from ten random families. All ten males were sequenced at the same facility and were from either the “Health/Medical/Biomedical” (HMB-IRB) or “Health/Medical/Biomedical” for non-profit organizations (HMB-IRB-NPU) consent groups, indicated as groups C1 and C2 in the ADSP pedigree files (available through dbGAP). We selected samples from the ADSP because we required samples that met the following criteria: (1) had been sequenced using standard paired-end Illumina sequencing with 100-nucleotide read lengths, (2) had been sequenced with a median depth > 30x, and (3) were publicly available. Median genome-wide read depths ranged from 33.0x to 45.0x coverage, with an overall median of 37.5x. Samples were prepared and sequenced as part of the ADSP [50]. These samples were aligned using BWA (v0.5.9). We could not find samples from the 1000 Genomes Project [24] that met these criteria; either sequencing depths were too shallow or read lengths were too long or short. The ADSP sample IDs we used were as follows: A-CUHS-CU000406, A-CUHS-CU002997, A-CUHS-CU000779, A-CUHS-CU000208, A-CUHS-CU001010, A-CUHS-CU002031, A-CUHS-CU002707, A-CUHS-CU003023, A-CUHS-CU003090, and A-CUHS-CU003128.

To assess dark and camouflaged regions in samples sequenced using Illumina 250-nucleotide read lengths, we selected ten samples from the 1000 Genomes Project that had been sequenced with 250-nucleotide read lengths and had a median depth > 30x. All ten samples were aligned using BWA (v 0.7.5a-r428) [2, 11–13]. Median genome-wide read depths ranged 30.0x to 61.0x coverage, with an overall median of 58.5x. Sample IDs for the Illumina 250-nucleotide read lengths were as follows: NA20845, HG01112, HG01583, HG01051, HG03742, HG00096, HG01565, HG01879, HG01500, and HG03006 (see Availability of Data and Materials section for public links).

We also selected samples generated using the 10x Genomics synthetic long-read sequencing platform and ONT and PacBio long-read sequencing platforms that were publicly available from the respective company. Specifically, we downloaded HG00512 raw FASTQ data from 10x Genomics and aligned it according to 10x Genomics’ standard practices. We used longranger (v2.2.2) and aligned to GRCh38 (longranger wgs --id HG00512 --description=“Han Chinese” --sex=“male” --fastqs=chi/HNKHFCCXX/,chi/HWHFTCCXX/ --reference=“10x-GRCh38-2.1.0/” --jobmode=sge --mempercore=125 –downsample=385). We also aligned to GRCh37. We were unable to align the 10x data to GRCh38+alt because longranger has a limit to the number of contigs it will align to. Median depth for HG00512 was 52x. For ONT, we downloaded the final Cliveome v3 from ONT’s official GitHub page (https://github.com/nanoporetech/ONT-HG1) and aligned it to GRCh37, GRCh38, and GRCh38+alt using minimap2 [104] (ALIGN_OPTS=“x map-pb -a --eqx -L -O 5,56 -E 4,1 -B 5 --secondary=no -z 400,50 -r 2k -Y”; REF=g1kv37/g1kv37.fa; minimap2 -d ${REF}.mmi ${ALIGN_OPTS} ${REF}; minimap2 ${ALIGN_OPTS} -a ${REF}.mmi <reads.fq> | samtools view -T {REF} -F 2308 > output_file). Cliveome v3 was sequenced to a median depth of 52x. We used the same alignment options recommended for PacBio because we found the recommended “map-ont” option in minimap2 performed substantially worse. We used PacBio data generated from HG005 [105], which was sequenced to a median depth of 50x and aligned using minimap2 [104] (pbsv fasta [movie].subreads.bam | minimap2 -t 8 -x map-pb -a --eqx -L -O 5,56 -E 4,1 -B 5 --secondary=no -z 400,50 -r 2 k -Y ftp://ftp.1000genomes.ebi.ac.uk/vol1/ftp/technical/reference/phase2_reference_assembly_sequence/hs37d5.fa.gz - | samtools sort > HG005_PacBio_GRCh38.bam). Neither the ONT nor the PacBio alignments included secondary alignments.

### Identifying dark and camouflaged gene body regions

To identify dark and camouflaged gene body regions in standard Illumina 100-nucleotide read length data, we first scanned all ten ADSP whole-genome sequence samples for genomic positions that met either of the following criteria: (1) had ≤ 5 reads and (2) had ≥ 90% of reads with a mapping quality (MAPQ) < 10. We then averaged the depth and count of low MAPQ reads across all samples for each position. We used strict cutoffs to identify regions that are clearly dark, but there are many additional regions that fall just beyond our thresholds. This analysis was performed using the Dark Region Finder (DRF; https://github.com/mebbert/DarkRegionFinder; mapq = 9; dark_mass=90; camo_mass=50; dark_depth=5; java -jar -Xmx20g DarkRegionFinder.jar -i <sample>.bam --human-ref genome.fa --min-region-size 1 --camo-mapq-threshold $mapq --min-dark-mapq-mass $dark_mass --min-camo-mapq-mass $camo_mass --dark-depth $dark_depth --camo-bed-output <sample>-camo-dark_depth_${dark_depth}-dark_mass_${dark_mass}-camo_mass_${camo_mass}-mapq_${mapq}.b38.bed --dark-bed-output <sample>-dark-dark_depth_${dark_depth}-dark_mass_${dark_mass}.b38.bed --incomplete-bed-output <sample>-incomplete.b38.bed). Any position that met either criterion was considered dark and categorized as either dark by depth or dark by mapping quality. For gene-body analyses, we then limited the dark regions to gene bodies by intersecting dark regions identified by Dark Region Finder with Ensembl’s GRCh37 build 87 or GRCh38 build 93 gene annotations. We converted the transcript-level annotations to gene-level annotations using bedtools [106] and custom scripts that are available. Any dark region that spanned a gene body element region (e.g., intron-exon boundary) was split into two separate dark regions so we could estimate the number of dark bases in each type of gene body region (e.g., introns, exons, UTRs). For all analyses, we only included dark regions with ≥ 20 contiguous bases. To identify camouflaged regions, specifically, we used BLAT [26] to identify all genomic regions that were highly similar to any given gene body region that was dark by mapping quality. Any region that was ≥ 98% identical (-minIdentity = 98), and that was considered dark (≥ 90% of reads with MAPQ < 10), was considered a match. We generated .bed files for all three genome builds using this method.

### Statistics

We quantified the percentage of each gene body that was dark by summing the total number of dark bases in the gene (i.e., between the 5′UTR to the 3′UTR start and end, respectively) and dividing by the total number of bases in the gene. We similarly calculated the percentage of intronic, exonic (including CDS and UTR), and only CDS exons by dividing the total number of dark bases in each category within the gene by the total number of bases within that category. We performed these calculations for data based on Illumina 100-nucleotide reads for all dark regions combined (Additional file 2: Table S1; Additional file 3: Table S2), dark by depth only (Additional file 15: Table S14; Additional file 16: Table S15), dark by mapping quality (Additional file 17: Table S16; Additional file 18: Table S17), and only camouflaged regions (Additional file 13: Table S12; Additional file 14: Table S13). We performed identical calculations for the samples from Illumina 250-nucleotide read length data, 10x Genomics, ONT, and PacBio (Additional file 4: Table S3; Additional file 5: Table S4; Additional file 6: Table S5; Additional file 7: Table S6; Additional file 8: Table S7; Additional file 9: Table S8; Additional file 10: Table S9; Additional file 11: Table S10 and Additional file 19: Table S18; Additional file 20: Table S19; Additional file 21: Table S20; Additional file 22: Table S21; Additional file 23: Table S22; Additional file 24: Table S23; Additional file 25: Table S24; Additional file 26: Table S25; Additional file 27: Table S26; Additional file 28: Table S27; Additional file 29: Table S28; Additional file 30: Table S29; Additional file 31: Table S30; Additional file 32: Table S31; Additional file 33: Table S32; Additional file 34: Table S33; Additional file 35: Table S34; Additional file 36: Table S35; Additional file 37: Table S36; Additional file 38: Table S37; Additional file 39: Table S38; Additional file 40: Table S39; Additional file 41: Table S40; Additional file 42: Table S41). We identified diseases that were known to be associated with genes that are at least 5% dark CDS by searching for mutations in the Human Gene Mutation Database (HGMD) [30]. For the area under the curve (AUC) comparison, we calculated the AUC for the Illumina 100-nucleotide data and normalized that to 1.0. The AUC is the sum of the percentage of dark nucleotides for each gene. The AUC for each other technology is represented as a proportion of the Illumina 100-nucleotide data.

Coverage plots from gnomAD data were obtained from gnomAD-old.broadinstitute.org [36]. We used the old version because the current version of gnomAD (accessed December 2018) does not allow the user to view median read depths, nor the percentage of samples with greater than a given coverage depth. Sequence pileups in representative samples were generated using the Integrative Genomics Viewer (IGV) [107], where reads with a MAPQ < 10 were filtered, and insertions, deletions, and mismatches were not shown. Karyotype plots showing genomic locations for dark and camouflaged regions were generated using KaryotypeR (v1.6.2) [108] in R (v3.5.1). Bar plots were made using ggplot2 (v3.0.0). Pathway analyses and resulting plots were generated using Metascape (accessed December 2018) [109]. Word clouds were generated at wordclouds.com. Gene schematics were generated using the Gene Structure Display Server (GSDS; v2) [110].

We performed an enrichment analysis to assess whether genes that are ≥ 5% dark CDS are enriched for specific diseases. Because we identified 76 genes that have a known mutation associated with disease, and that are ≥ 5% dark CDS, we randomly selected 76 genes from the known HGMD mutations and measured the number of genes with known mutation associated with each disease. We repeated this process 10,000 times and used the following metric as our enrichment score: − 10*log10(empirical_pvalue), rounded to the nearest whole number.

### Screening ADSP for functional *CR1* mutations in camouflaged region

After discovering that 26% of the *CR1* gene’s CDS is camouflaged, we screened all ADSP samples for rare functional mutations that could play a role in Alzheimer’s disease development and progression by applying our proposed method (Fig. 8). To apply our method, we extracted all reads with a mapping quality (MAPQ) < 10 from each camouflaged region within *CR1*, and from each of the respective camouflage mate regions, using samtools and the GRCh38 .bed file we generated that identifies all camouflaged regions. An example of camouflaged mate regions in *CR1* includes exons 10, 18, and 26, which are identical in the reference genome (Fig. 8). As previously mentioned, *CR1* is a special case that is camouflaged by regions duplicated within itself, rather than being camouflaged by a different gene; thus, we knew that any mutations we discovered would be from *CR1*. Our approach works the same regardless of whether a gene is camouflaged by itself or another gene, but we mention that *CR1* is camouflaged by itself, for interest. After extracting reads from each camouflaged region, using the .bed file we provide, we then masked all camouflaged regions within *CR1* in the reference genome, except for one from each set of camouflaged mates. For example, between exons 10, 18, and 26, we masked exons 18 and 26 in the reference genome, allowing reads from all three exons to align only to exon 10; without competing camouflaged regions to confuse the aligner, all reads from exons 10, 18, and 26 mapped to exon 10 with high quality. Masking regions of the reference genome simply means to change nucleotides to an unmappable character (usually “N”), to prevent any reads from aligning to that region.

After aligning all reads to a single region within each set of camouflaged regions, we were able to perform standard variant calling using the GATK HaplotypeCaller [25], with two exceptions: (1) instead of treating each camouflaged region as diploid, we increased the ploidy setting in HaplotypeCaller according to the number of copies within a given set of camouflaged regions, and (2) we filtered all reference-based artifacts. Reference-based artifacts arise from aligning reads from two non-identical regions to a single region, causing false-positive mutations. Referring again to our *CR1* example, because there are three regions (exons 10, 18, and 26), we set the HaplotypeCaller ploidy to hexaploid. Increasing the ploidy is essential for increased sensitivity, since the number of reads harboring a given variant—which only originate from one of the camouflaged regions—will be overwhelmed by reads from the others, thus preventing the variant caller from identifying the mutation under the assumption that the data are from a diploid region. In other words, if a mutation exists in exon 26, we would expect only approximately 1/6th of reads from exons 10, 18, and 26 to harbor that mutation, rather than approximately 1/2. Because the ADSP is mostly exome data, we limited HaplotypeCaller to CDS exons only. According to the current ADSP phenotype data, one of the samples harboring the *CR1* frameshift mutation is a control. The individual has since been officially diagnosed with Alzheimer’s disease, however. We used KING-robust to determine kinship between individuals [51].

To identify reference-based artifacts, all camouflaged CDS regions repeated ≤ 5 times were blatted against the whole genome. DNA sequence from hits with at least 98% sequence identity was locally aligned back to the query sequence. Bio.pairwise2 module in Biopython was used for local alignments using following parameters: match = 1, mismatch = − 3, gapOpen = − 5, gapExtend = − 2. Mismatches or gaps in the resulting aligned sequence were converted into variant positions based on the start position of the query sequence in the genome and the position of the variant within the aligned sequence. Three hundred ninety-one reference-based artifact positions were found using this method. While running our pipeline to rescue variants, any variant called by GATK at one of these positions was filtered out.

## Additional files

Additional file 1: Supplemental figures. (DOCX 3853 kb)

Additional file 2: IlluminaRL100 percent dark genes in hg38. (TXT 401 kb)

Additional file 3: IlluminaRL100 dark gene annotations in hg38. (TXT 3152 kb)

Additional file 4: IlluminaRL250 percent dark genes in hg38. (TXT 282 kb)

Additional file 5: IlluminaRL250 dark gene annotations in hg38. (TXT 1798 kb)

Additional file 6: ONT percent dark genes in hg38. (TXT 291 kb)

Additional file 7: ONT dark gene annotations in hg38. (TXT 732 kb)

Additional file 8: PacBio percent dark genes in hg38. (TXT 145 kb)

Additional file 9: PacBio dark gene annotations in hg38. (TXT 759 kb)

Additional file 10: 10x Genomics percent dark genes in hg38. (TXT 262 kb)

Additional file 11: 10x Genomics dark gene annotations in hg38. (TXT 1247 kb)

Additional file 12: Gene Ontology results for dark genes in IlluminaRL100 for hg38. (CSV 47 kb)

Additional file 13: IlluminaRL100 percent camouflaged genes in hg38. (TXT 183 kb)

Additional file 14: IlluminaRL100 camouflaged gene annotations in hg38. (TXT 2141 kb)

Additional file 15: IlluminaRL100 percent dark-by-depth genes in hg38. (TXT 241 kb)

Additional file 16: IlluminaRL100 dark-by-depth gene annotations in hg38. (TXT 647 kb)

Additional file 17: IlluminaRL100 percent dark-by-MAPQ genes in hg38. (TXT 253 kb)

Additional file 18: IlluminaRL100 dark-by-MAPQ gene annotations in hg38. (TXT 2710 kb)

Additional file 19: IlluminaRL250 percent camouflaged genes in hg38. (TXT 142 kb)

Additional file 20: IlluminaRL250 camouflaged gene annotations in hg38. (TXT 1279 kb)

Additional file 21: IlluminaRL250 percent dark-by-depth genes in hg38. (TXT 147 kb)

Additional file 22: IlluminaRL250 dark-by-depth gene annotations in hg38. (TXT 354 kb)

Additional file 23: IlluminaRL250 percent dark-by-MAPQ genes in hg38. (TXT 188 kb)

Additional file 24: IlluminaRL250 dark-by-MAPQ gene annotations in hg38. (TXT 1574 kb)

Additional file 25: ONT percent camouflaged genes in hg38. (TXT 13 kb)

Additional file 26: ONT camouflaged gene annotations in hg38. (TXT 122 kb)

Additional file 27: ONT percent dark-by-depth genes in hg38. (TXT 278 kb)

Additional file 28: ONT dark-by-depth gene annotations in hg38. (TXT 556 kb)

Additional file 29: ONT percent dark-by-MAPQ genes in hg38. (TXT 17 kb)

Additional file 30: ONT dark-by-MAPQ gene annotations in hg38. (TXT 210 kb)

Additional file 31: PacBio percent camouflaged genes in hg38. (TXT 31 kb)

Additional file 32: PacBio camouflaged gene annotations in hg38. (TXT 348 kb)

Additional file 33: PacBio percent dark-by-depth genes in hg38. (TXT 113 kb)

Additional file 34: PacBio dark-by-depth gene annotations in hg38. (TXT 334 kb)

Additional file 35: PacBio percent dark-by-MAPQ genes in hg38. (TXT 41 kb)

Additional file 36: PacBio dark-by-MAPQ gene annotations in hg38. (TXT 477 kb)

Additional file 37: 10x Genomics percent camouflaged genes in hg38. (TXT 55 kb)

Additional file 38: 10x Genomics camouflaged gene annotations in hg38. (TXT 459 kb)

Additional file 39: 10x Genomics percent dark-by-depth genes in hg38. (TXT 211 kb)

Additional file 40: 10x Genomics dark-by-depth gene annotations in hg38. (TXT 693 kb)

Additional file 41: 10x Genomics percent dark-by-MAPQ genes in hg38. (TXT 111 kb)

Additional file 42: 10x Genomics dark-by-MAPQ gene annotations in hg38. (TXT 730 kb)

## Abbreviations

ADSP: Alzheimer’s Disease Sequencing Project
ALS: Amyotrophic lateral sclerosis
CDS: Coding sequence
FTD: Frontotemporal dementia
MAPQ: Mapping quality
ONT: Oxford Nanopore Technologies
PacBio: Pacific Biosciences
