# Supplementary material for: Systematic analysis of dark and camouflaged genes reveals disease-relevant genes hiding in plain sight
Source: Genome Biol. 2019 May 20;20:97. doi: 10.1186/s13059-019-1707-2 (PMC6526621; doi:10.1186/s13059-019-1707-2)
Supplement: Supplementary file 1 — Supplemental figures. (DOCX 3853 kb) [file 13059_2019_1707_MOESM1_ESM.docx]

**Figure S1. Dark regions are scattered throughout the genome, but largely resolved by long-read sequencing technologies.** **(a)** We identified 36794 dark regions (>15 million nucleotides) in 6054 gene bodies (based on Ensemble GRCh38 build 93 gene annotations) that were either dark by depth or dark by mapping quality (Supplemental Tables 1-2). **(b)** Data from samples sequenced using 250-nucleotide Illumina read lengths reduced the area under the curve (AUC) by 12.2% for all gene bodies (Supplemental Tables 3-4). Comparing long-read sequencing technologies to the standard Illumina 100-nucleotide read lengths, PacBio, 10x Genomics, and ONT reduced the AUC by approximately 49.5%, 64.3%, and 77.0% for all gene bodies, respectively (Supplemental Tables 5-10). The ONT platform performed best, overall, reducing the total percentage of dark regions by 77.0% to 23.0%, for all gene bodies. **(c)** Long-read technologies improve upon short-read data primarily by reducing the percentage of regions that are dark by mapping quality.

**Figure S2. Most camouflaged regions are duplicated 2-3 times.** **(a)** We measured the number of times each gene region was duplicated and found that 71.1% of gene regions were replicated three or fewer times in the genome, but 42 regions were duplicated ≥100 times, with the most repeated region (intronic region from *FGF12* intron six) being replicated 530 times. **(b)** Limiting to only CDS regions, we estimate that 76.2% are replicated three or fewer times, with 45 replicated ≥10 times and the most repeated region was from NBPF20, which was replicated 32 times (Supplemental Figure 2b).


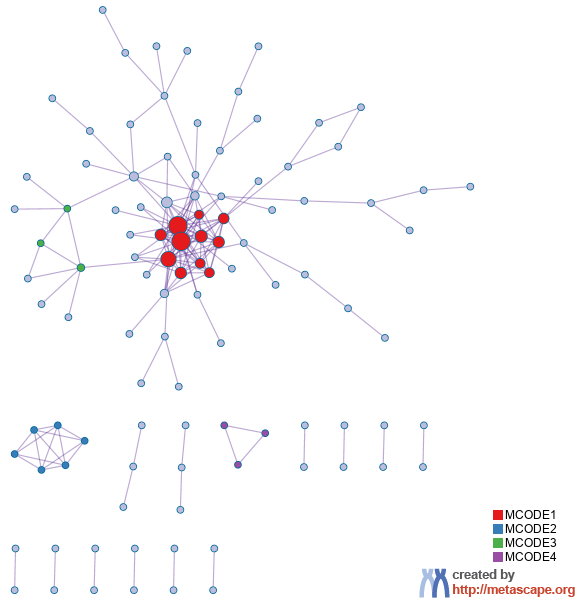


**Figure S3. There are 172 known protein-protein interactions amongst 103 dark genes.** Looking specifically at known protein-protein interactions, we found 103 proteins with 172 known interactions.

**
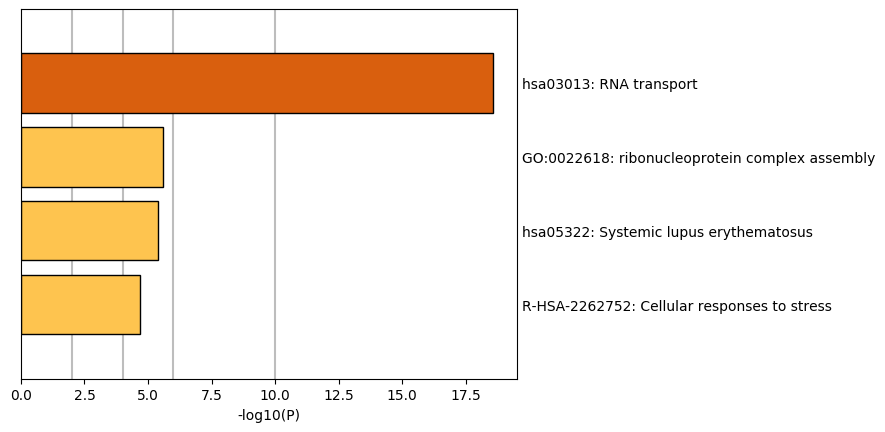
**

**Figure S4. All four MCODE groups combined are primarily associated with RNA transport.**

**
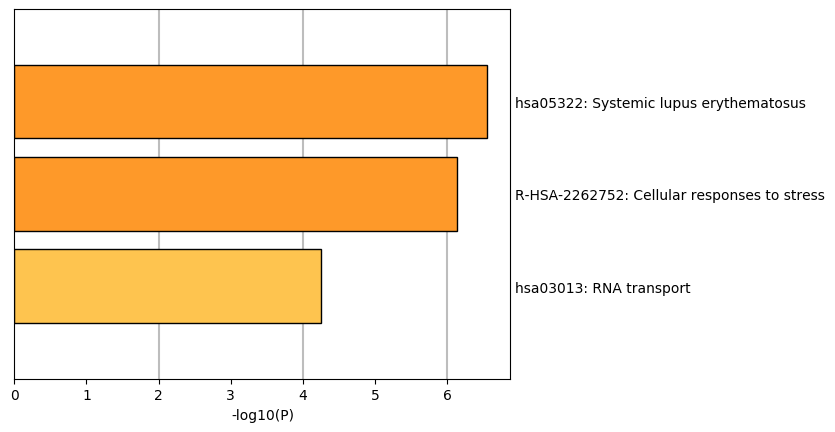
**

**Figure S5. MCODE group 1 is enriched for systemic lupus erythematosus and cellular responses to stress.**


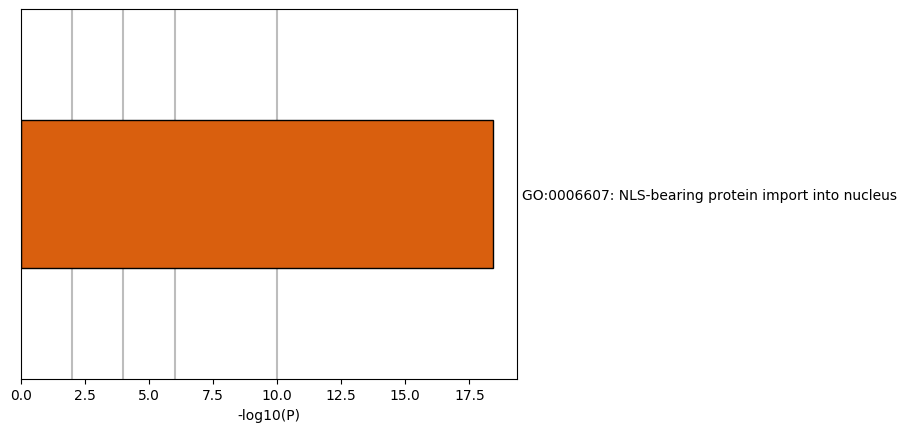


**Figure S6. MCODE group 2 is enriched with proteins involved in NLS-bearing protein import into nucleus and protein import into nucleus**.


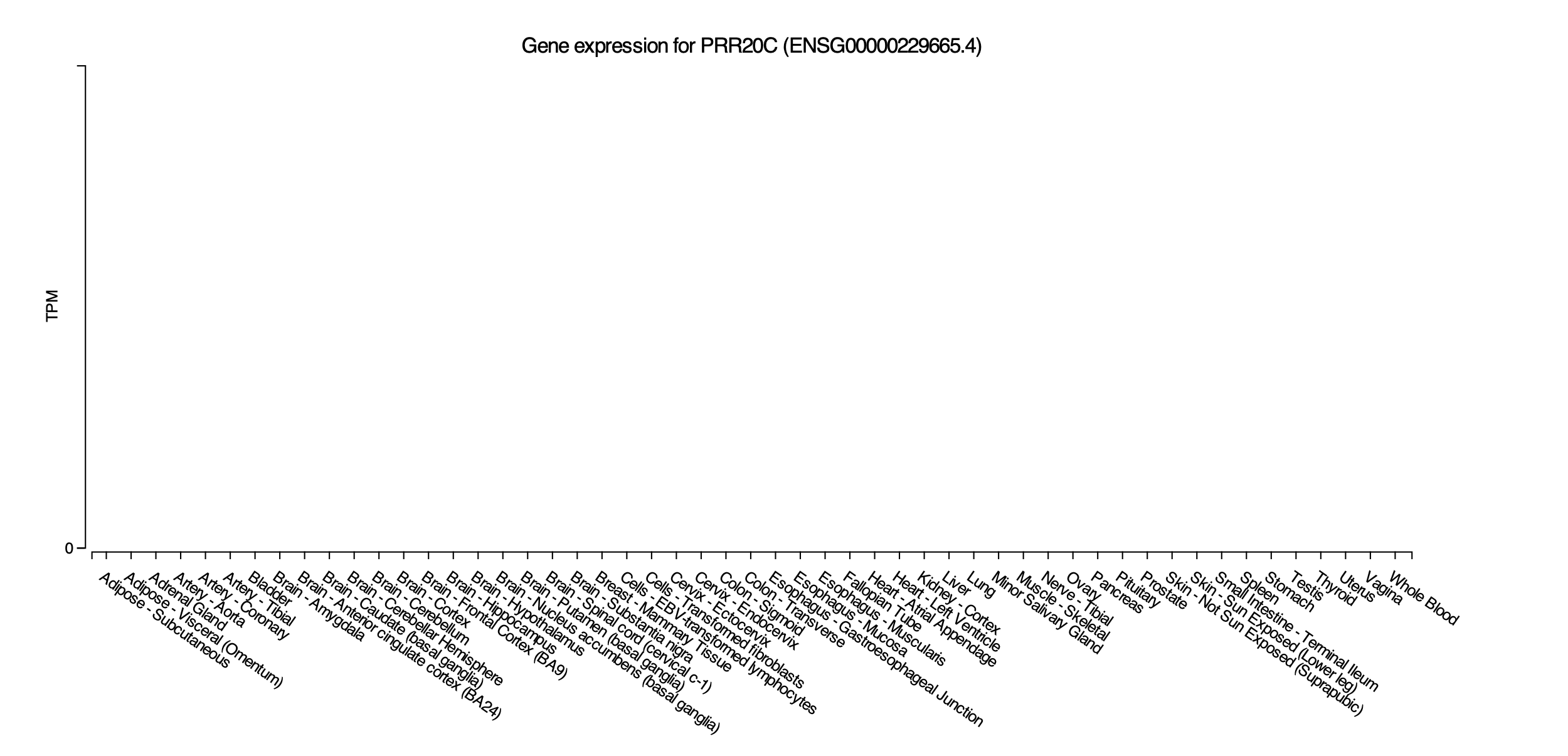


**Figure S7. PRR20C is 100% camouflaged and has no known expression in GTEx (accessed December 2018).**


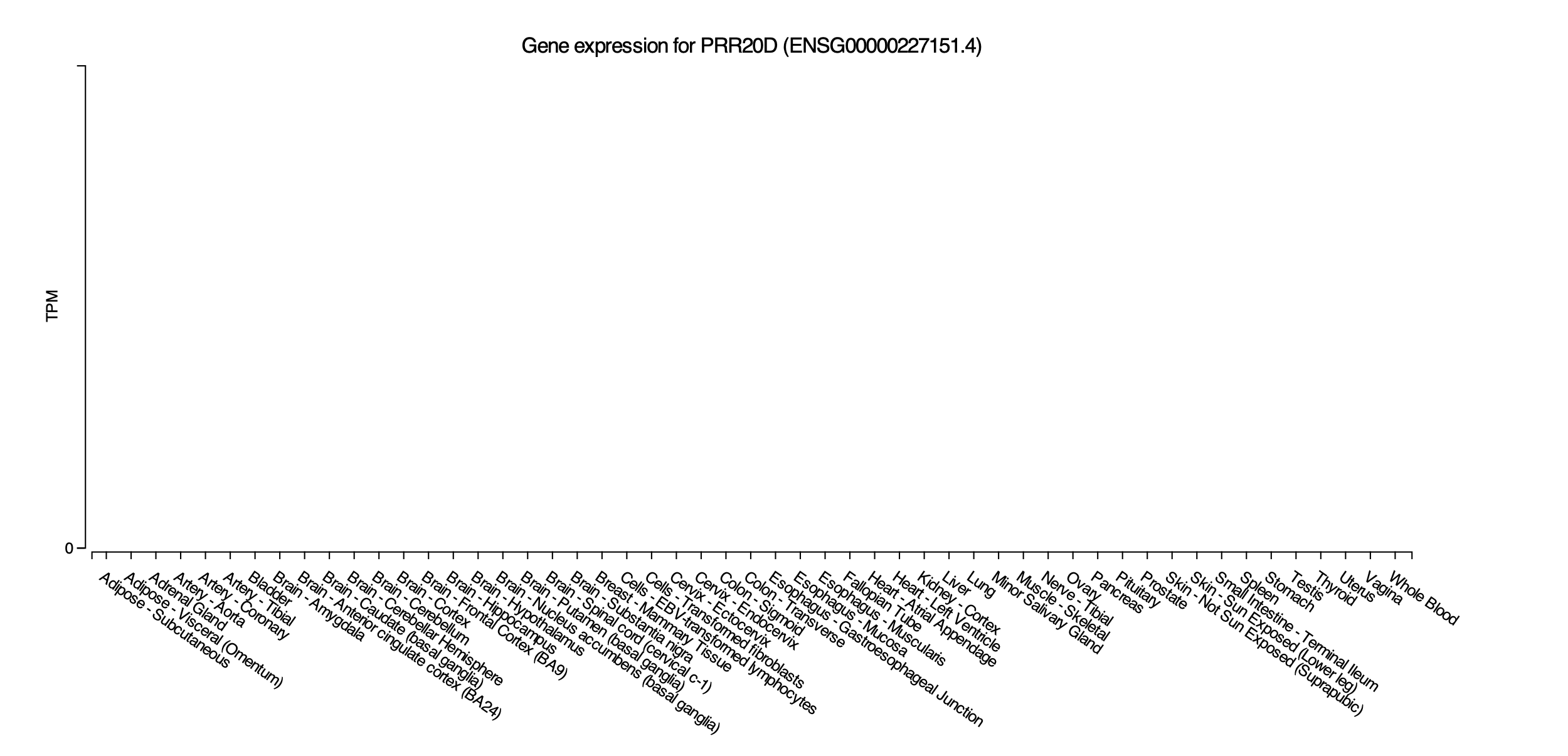


**Figure S8. PRR20D is 100% camouflaged and has no known expression in GTEx (accessed December 2018).**


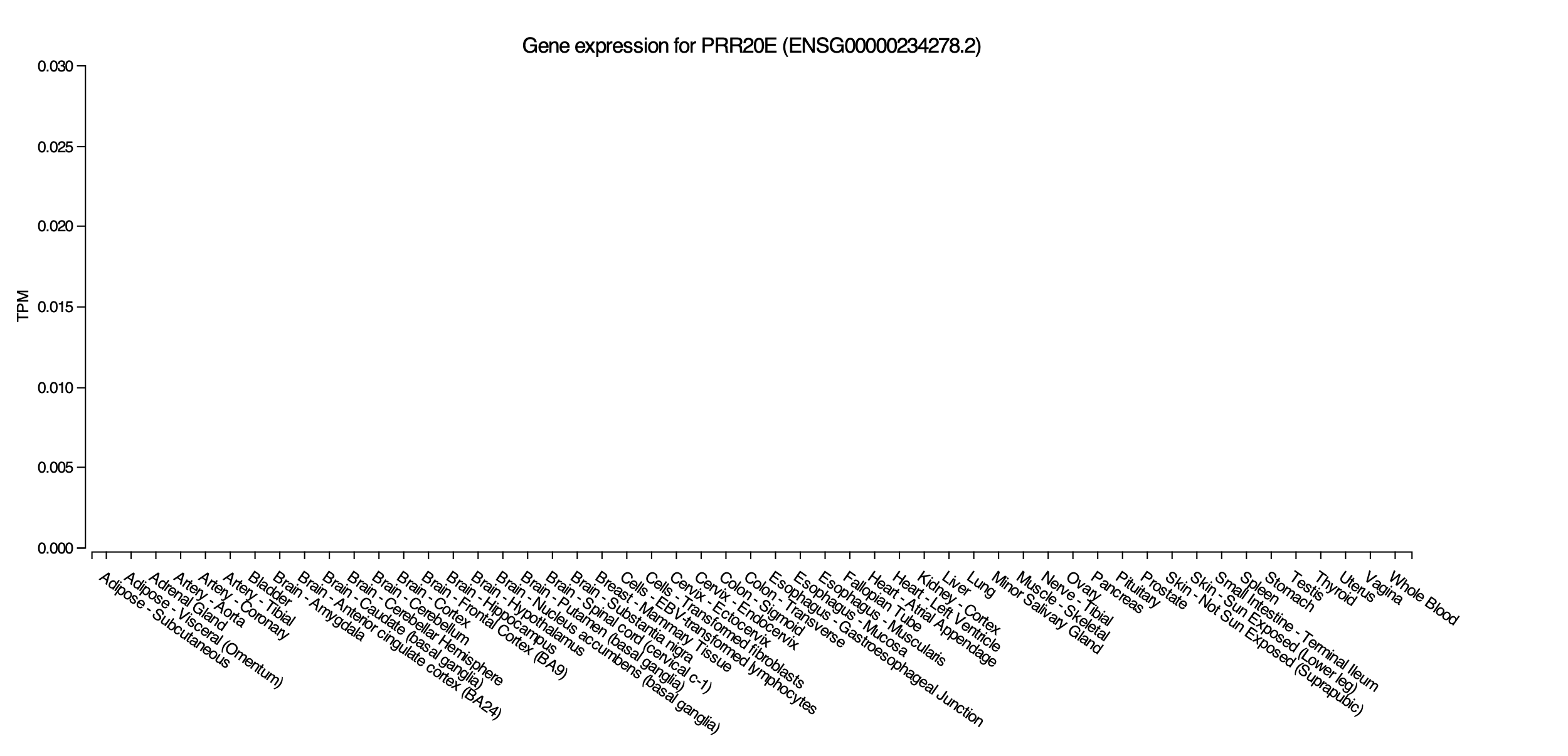


**Figure S9. PRR20E is 100% camouflaged and has no known expression in GTEx (accessed December 2018).**


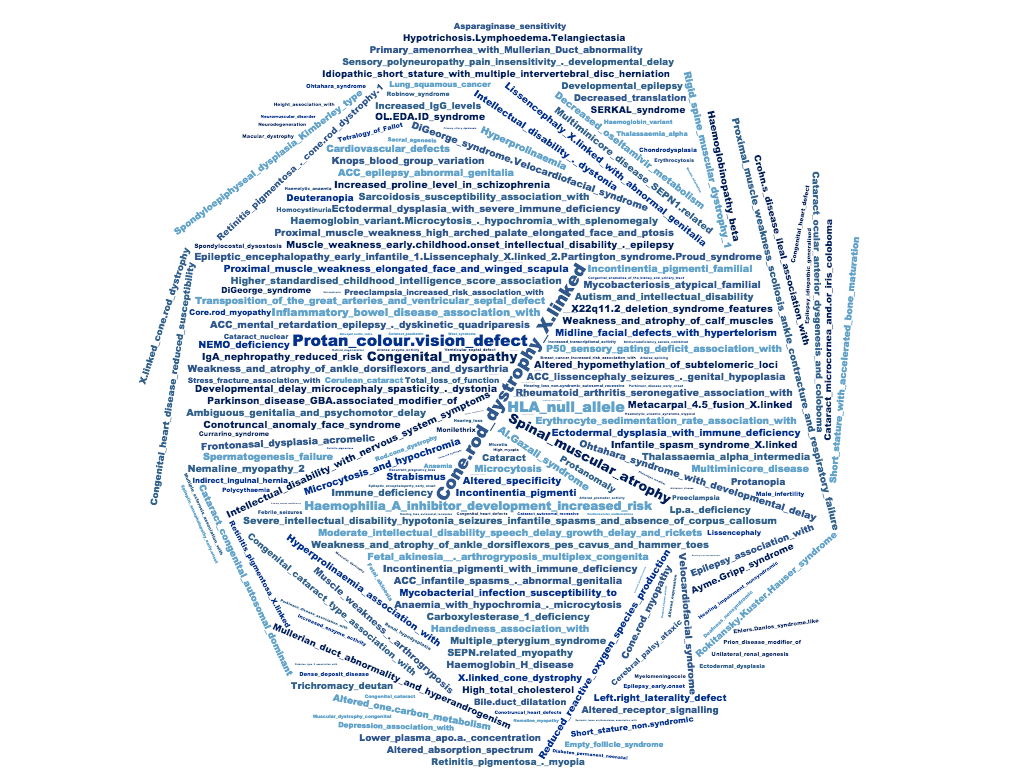


**Figure S10. Dark genes are enriched for genes involved in several diseases, including color blindness (protan colour vision defect), X-linked cone-rod dystrophy, and spinal muscular atrophy.** We performed an enrichment analysis, where the diseases most enriched for dark genes included color blindness (protan colour vision defect), X-linked cone-rod dystrophy, and spinal muscular atrophy.

**APOE**

**
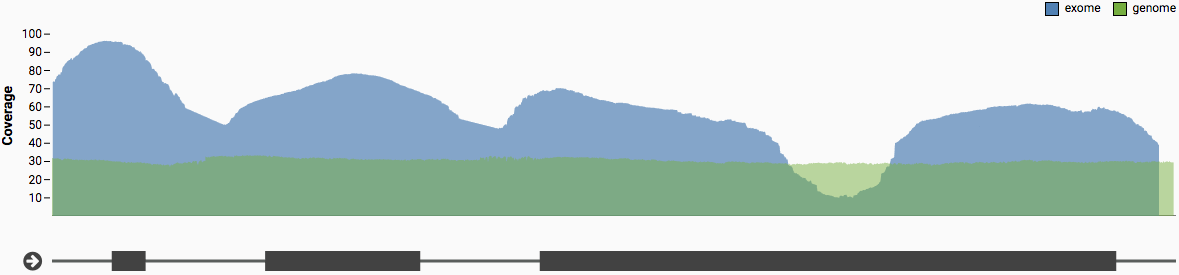
**

**Figure S11. The *APOE* gene is 6% dark in whole-exome data, and is dark for some samples with whole-genome sequencing.** *APOE*—the top genetic risk for Alzheimer’s disease—is approximately 6% dark CDS (by depth) for certain ADSP samples with whole-genome sequencing, and the same region is dark in gnomAD whole-exome data.

**
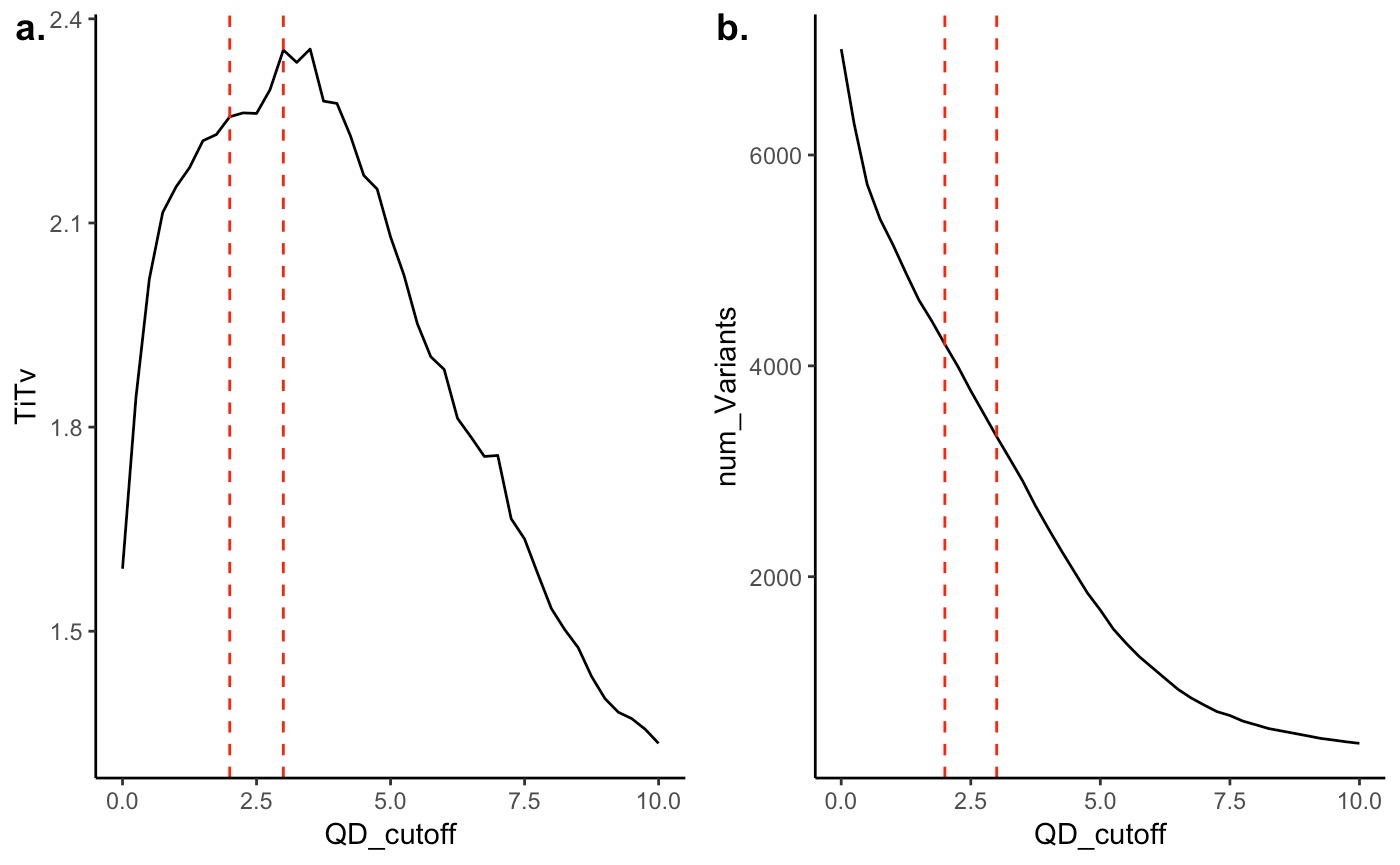
**

**Figure S12. Our method rescues between approximately 3152 and 4622 variants in 13142 ADSP samples.** Across 10933 samples from the ADSP, excluding all variants with a quality by depth (QD) ≤2, were able to rescue approximately 4214 exonic variants with a transition-transversion ration (Ti/Tv) of 2.26 from 137 camouflaged region sets, that are spread across 748 camouflaged genes (Supplemental File 1). Using more stringent QD (excluding variants with QD ≤3), we rescued 3343 variants with a Ti/Tv ratio of 2.35. We only included exons from genes that are at least 5% dark CDS. **(a)** We plotted Ti/Tv ratios versus QD cutoff. The Ti/Tv ratio at QD = 2.0 and QD = 3.0 are 2.26 and 2.35, respectively. **(b)** The number of variants rescued at QD = 2.0 and QD = 3.0 are 4214 and 3343, respectively.
